# Supplementary material for: Iron and copper on Botrytis cinerea: new inputs in the cellular characterization of their inhibitory effect
Source: PeerJ. 2023 Sep 20;11:e15994. doi: 10.7717/peerj.15994 (PMC10517660; doi:10.7717/peerj.15994)
Supplement: Supplemental Information 1 [file peerj-11-15994-s001.zip › Raw data/Adhesion of metals Statistics analysis.rtf]

Summary Statistics
	Count	Average	Median	Mode	Geometric mean	5% Trimmed mean	5% Winsorized mean	
Cu	3	0,626322	0,905254		0,336411	0,641818	0,626322	
Cufe	2	3,16734	3,16734	2,67155	3,12829	3,16734	3,16734	
Total	5	1,64273	0,928411		0,820814	1,61923	1,64273	

	Variance	Standard deviation	Coeff. of variation	Standard error	5% Winsorized sigma	
Cu	0,253324	0,503313	80,3601%	0,290588	0,503313	
Cufe	0,491604	0,701145	22,1367%	0,495784	0,701145	
Total	2,18659	1,47871	90,0157%	0,6613	1,47871	

	MAD	Sbi	Minimum	Maximum	Range	Lower quartile	Upper quartile	
Cu	0,0231577	0,283327	0,0453	0,928411	0,883111	0,0453	0,928411	
Cufe	0,495784	0,521878	2,67155	3,66312	0,991569	2,67155	3,66312	
Total	0,883111	1,55313	0,0453	3,66312	3,61782	0,905254	2,67155	

	Interquartile range	1/6 sextile	5/6 sextile	Intersextile range	Skewness	Stnd. skewness	Kurtosis	
Cu	0,883111	0,0453	0,928411	0,883111	-1,72793	-1,22183		
Cufe	0,991569	2,67155	3,66312	0,991569				
Total	1,7663	0,0453	3,66312	3,61782	0,579171	0,528708	-1,50298	

	Stnd. kurtosis	Sum	Sum of squares	
Cu		1,87896	1,68348	
Cufe		6,33467	20,5556	
Total	-0,686013	8,21364	22,2391	

The StatAdvisor
This table shows various statistics for each of the 2 columns of data.  To test for significant differences amongst the column means, select Analysis of Variance from the list of Tabular Options.  Select Means Plot from the list of Graphical Options to display the means graphically.  


ANOVA Table
Source	Sum of Squares	Df	Mean Square	F-Ratio	P-Value	
Between groups	7,7481	1	7,7481	23,29	0,0170	
Within groups	0,998251	3	0,33275			
Total (Corr.)	8,74635	4				

The StatAdvisor
The ANOVA table decomposes the variance of the data into two components: a between-group component and a within-group component.  The F-ratio, which in this case equals 23,285, is a ratio of the between-group estimate to the within-group estimate.  Since the P-value of the F-test is less than 0,05, there is a statistically significant difference between the means of the 2 variables at the 95,0% confidence level.  To determine which means are significantly different from which others, select Multiple Range Tests from the list of Tabular Options.

Table of Means with 95,0 percent LSD intervals
			Stnd. error			
	Count	Mean	(pooled s)	Lower limit	Upper limit	
Cu	3	0,626322	0,333042	-0,123133	1,37578	
Cufe	2	3,16734	0,407891	2,24944	4,08523	
Total	5	1,64273				

The StatAdvisor
This table shows the mean for each column of data.  It also shows the standard error of each mean, which is a measure of its sampling variability.  The standard error is formed by dividing the pooled standard deviation by the square root of the number of observations at each level.  The table also displays an interval around each mean.  The intervals currently displayed are based on Fisher's least significant difference (LSD) procedure.  They are constructed in such a way that if two means are the same, their intervals will overlap 95,0% of the time.  You can display the intervals graphically by selecting Means Plot from the list of Graphical Options.  In the Multiple Range Tests, these intervals are used to determine which means are significantly different from which others.

Multiple Range Tests

Method: 95,0 percent LSD
	Count	Mean	Homogeneous Groups	
Cu	3	0,626322	X	
Cufe	2	3,16734	 X	

Contrast	Sig.	Difference	+/- Limits	
Cu - Cufe	 *	-2,54101	1,67583	
* denotes a statistically significant difference.

The StatAdvisor
This table applies a multiple comparison procedure to determine which means are significantly different from which others.  The bottom half of the output shows the estimated difference between each pair of means.  An asterisk has been placed next to 1 pair, indicating that this pair shows a statistically significant difference at the 95,0% confidence level.  At the top of the page, 2 homogenous groups are identified using columns of X's.  Within each column, the levels containing X's form a group of means within which there are no statistically significant differences.  The method currently being used to discriminate among the means is Fisher's least significant difference (LSD) procedure.  With this method, there is a 5,0% risk of calling each pair of means significantly different when the actual difference equals 0.  

Multiple-Sample Comparison
Sample 1: Cufe
Sample 2: Cu

Sample 1: 2 values ranging from 2,67155 to 3,66312
Sample 2: 3 values ranging from 0,0453 to 0,928411

The StatAdvisor
This procedure compares the data in 2 columns of the current data file.  It constructs various statistical tests and graphs to compare the samples.  The F-test in the ANOVA table will test whether there are any significant differences amongst the means.  If there are, the Multiple Range Tests will tell you which means are significantly different from which others.  If you are worried about the presence of outliers, choose the Kruskal-Wallis Test which compares medians instead of means.  The various plots will help you judge the practical significance of the results, as well as allow you to look for possible violations of the assumptions underlying the analysis of variance.  


Summary Statistics
	Count	Average	Median	Mode	Geometric mean	5% Trimmed mean	5% Winsorized mean	
Cufe	2	3,16734	3,16734	2,67155	3,12829	3,16734	3,16734	
Cu	3	0,626322	0,905254		0,336411	0,641818	0,626322	
Total	5	1,64273	0,928411		0,820814	1,61923	1,64273	

	Variance	Standard deviation	Coeff. of variation	Standard error	5% Winsorized sigma	
Cufe	0,491604	0,701145	22,1367%	0,495784	0,701145	
Cu	0,253324	0,503313	80,3601%	0,290588	0,503313	
Total	2,18659	1,47871	90,0157%	0,6613	1,47871	

	MAD	Sbi	Minimum	Maximum	Range	Lower quartile	Upper quartile	
Cufe	0,495784	0,521878	2,67155	3,66312	0,991569	2,67155	3,66312	
Cu	0,0231577	0,283327	0,0453	0,928411	0,883111	0,0453	0,928411	
Total	0,883111	1,55313	0,0453	3,66312	3,61782	0,905254	2,67155	

	Interquartile range	1/6 sextile	5/6 sextile	Intersextile range	Skewness	Stnd. skewness	Kurtosis	
Cufe	0,991569	2,67155	3,66312	0,991569				
Cu	0,883111	0,0453	0,928411	0,883111	-1,72793	-1,22183		
Total	1,7663	0,0453	3,66312	3,61782	0,579171	0,528708	-1,50298	

	Stnd. kurtosis	Sum	Sum of squares	
Cufe		6,33467	20,5556	
Cu		1,87896	1,68348	
Total	-0,686013	8,21364	22,2391	

The StatAdvisor
This table shows various statistics for each of the 2 columns of data.  To test for significant differences amongst the column means, select Analysis of Variance from the list of Tabular Options.  Select Means Plot from the list of Graphical Options to display the means graphically.  


ANOVA Table
Source	Sum of Squares	Df	Mean Square	F-Ratio	P-Value	
Between groups	7,7481	1	7,7481	23,29	0,0170	
Within groups	0,998251	3	0,33275			
Total (Corr.)	8,74635	4				

The StatAdvisor
The ANOVA table decomposes the variance of the data into two components: a between-group component and a within-group component.  The F-ratio, which in this case equals 23,285, is a ratio of the between-group estimate to the within-group estimate.  Since the P-value of the F-test is less than 0,05, there is a statistically significant difference between the means of the 2 variables at the 95,0% confidence level.  To determine which means are significantly different from which others, select Multiple Range Tests from the list of Tabular Options.

Multiple Range Tests

Method: 95,0 percent LSD
	Count	Mean	Homogeneous Groups	
Cu	3	0,626322	X	
Cufe	2	3,16734	 X	

Contrast	Sig.	Difference	+/- Limits	
Cufe - Cu	 *	2,54101	1,67583	
* denotes a statistically significant difference.

The StatAdvisor
This table applies a multiple comparison procedure to determine which means are significantly different from which others.  The bottom half of the output shows the estimated difference between each pair of means.  An asterisk has been placed next to 1 pair, indicating that this pair shows a statistically significant difference at the 95,0% confidence level.  At the top of the page, 2 homogenous groups are identified using columns of X's.  Within each column, the levels containing X's form a group of means within which there are no statistically significant differences.  The method currently being used to discriminate among the means is Fisher's least significant difference (LSD) procedure.  With this method, there is a 5,0% risk of calling each pair of means significantly different when the actual difference equals 0.  

Multiple-Sample Comparison
Sample 1: Fe
Sample 2: Cu

Sample 1: 2 values ranging from 2,70071 to 3,97891
Sample 2: 3 values ranging from 0,0453 to 0,928411

The StatAdvisor
This procedure compares the data in 2 columns of the current data file.  It constructs various statistical tests and graphs to compare the samples.  The F-test in the ANOVA table will test whether there are any significant differences amongst the means.  If there are, the Multiple Range Tests will tell you which means are significantly different from which others.  If you are worried about the presence of outliers, choose the Kruskal-Wallis Test which compares medians instead of means.  The various plots will help you judge the practical significance of the results, as well as allow you to look for possible violations of the assumptions underlying the analysis of variance.  


Table of Means with 95,0 percent LSD intervals
			Stnd. error			
	Count	Mean	(pooled s)	Lower limit	Upper limit	
Fe	2	3,33981	0,469669	2,2829	4,39672	
Cu	3	0,626322	0,383483	-0,236643	1,48929	
Total	5	1,71172				

The StatAdvisor
This table shows the mean for each column of data.  It also shows the standard error of each mean, which is a measure of its sampling variability.  The standard error is formed by dividing the pooled standard deviation by the square root of the number of observations at each level.  The table also displays an interval around each mean.  The intervals currently displayed are based on Fisher's least significant difference (LSD) procedure.  They are constructed in such a way that if two means are the same, their intervals will overlap 95,0% of the time.  You can display the intervals graphically by selecting Means Plot from the list of Graphical Options.  In the Multiple Range Tests, these intervals are used to determine which means are significantly different from which others.

Multiple-Sample Comparison
Sample 1: Fe
Sample 2: Cufe

Sample 1: 2 values ranging from 2,70071 to 3,97891
Sample 2: 2 values ranging from 2,67155 to 3,66312

The StatAdvisor
This procedure compares the data in 2 columns of the current data file.  It constructs various statistical tests and graphs to compare the samples.  The F-test in the ANOVA table will test whether there are any significant differences amongst the means.  If there are, the Multiple Range Tests will tell you which means are significantly different from which others.  If you are worried about the presence of outliers, choose the Kruskal-Wallis Test which compares medians instead of means.  The various plots will help you judge the practical significance of the results, as well as allow you to look for possible violations of the assumptions underlying the analysis of variance.  


ANOVA Table
Source	Sum of Squares	Df	Mean Square	F-Ratio	P-Value	
Between groups	0,0297474	1	0,0297474	0,05	0,8509	
Within groups	1,30849	2	0,654246			
Total (Corr.)	1,33824	3				

The StatAdvisor
The ANOVA table decomposes the variance of the data into two components: a between-group component and a within-group component.  The F-ratio, which in this case equals 0,0454682, is a ratio of the between-group estimate to the within-group estimate.  Since the P-value of the F-test is greater than or equal to 0,05, there is not a statistically significant difference between the means of the 2 variables at the 95,0% confidence level.

Table of Means with 95,0 percent LSD intervals
			Stnd. error			
	Count	Mean	(pooled s)	Lower limit	Upper limit	
Fe	2	3,33981	0,571947	1,5997	5,07992	
Cufe	2	3,16734	0,571947	1,42722	4,90745	
Total	4	3,25357				

The StatAdvisor
This table shows the mean for each column of data.  It also shows the standard error of each mean, which is a measure of its sampling variability.  The standard error is formed by dividing the pooled standard deviation by the square root of the number of observations at each level.  The table also displays an interval around each mean.  The intervals currently displayed are based on Fisher's least significant difference (LSD) procedure.  They are constructed in such a way that if two means are the same, their intervals will overlap 95,0% of the time.  You can display the intervals graphically by selecting Means Plot from the list of Graphical Options.  In the Multiple Range Tests, these intervals are used to determine which means are significantly different from which others.

Multiple Range Tests

Method: 95,0 percent LSD
	Count	Mean	Homogeneous Groups	
Cufe	2	3,16734	X	
Fe	2	3,33981	X	

Contrast	Sig.	Difference	+/- Limits	
Fe - Cufe		0,172474	3,48022	
* denotes a statistically significant difference.

The StatAdvisor
This table applies a multiple comparison procedure to determine which means are significantly different from which others.  The bottom half of the output shows the estimated difference between each pair of means.  There are no statistically significant differences between any pair of means at the 95,0% confidence level.  At the top of the page, one homogenous group is identified by a column of X's.  Within each column, the levels containing X's form a group of means within which there are no statistically significant differences.  The method currently being used to discriminate among the means is Fisher's least significant difference (LSD) procedure.  With this method, there is a 5,0% risk of calling each pair of means significantly different when the actual difference equals 0.  
