# Supplementary material for: Iron and copper on Botrytis cinerea: new inputs in the cellular characterization of their inhibitory effect
Source: PeerJ. 2023 Sep 20;11:e15994. doi: 10.7717/peerj.15994 (PMC10517660; doi:10.7717/peerj.15994)
Supplement: Supplemental Information 1 [file peerj-11-15994-s001.zip › Raw data/CR Cu Statistics analysis.rtf]

Multiple-Sample Comparison
Sample 1: CR
Sample 2: Cu

Sample 1: 9 values ranging from 61,9 to 72,0
Sample 2: 6 values ranging from 61,35 to 69,3

The StatAdvisor
This procedure compares the data in 2 columns of the current data file.  It constructs various statistical tests and graphs to compare the samples.  The F-test in the ANOVA table will test whether there are any significant differences amongst the means.  If there are, the Multiple Range Tests will tell you which means are significantly different from which others.  If you are worried about the presence of outliers, choose the Kruskal-Wallis Test which compares medians instead of means.  The various plots will help you judge the practical significance of the results, as well as allow you to look for possible violations of the assumptions underlying the analysis of variance.  


Summary Statistics
	Count	Average	Median	Mode	Geometric mean	5% Trimmed mean	5% Winsorized mean	
CR	9	65,8033	65,82		65,7393	65,6759	65,8033	
Cu	6	64,515	63,95		64,4553	64,425	64,515	
Total	15	65,288	64,4	64,4	65,2226	65,1339	65,288	

	Variance	Standard deviation	Coeff. of variation	Standard error	5% Winsorized sigma	MAD	
CR	9,6598	3,10802	4,7232%	1,03601	3,10802	2,11	
Cu	9,37575	3,06198	4,74616%	1,25005	3,06198	2,405	
Total	9,29517	3,0488	4,66977%	0,787196	3,0488	2,4	

	Sbi	Minimum	Maximum	Range	Lower quartile	Upper quartile	Interquartile range	
CR	2,96984	61,9	72,0	10,1	63,71	67,0	3,29	
Cu	2,91622	61,35	69,3	7,95	61,74	66,8	5,06	
Total	3,05358	61,35	72,0	10,65	62,7	67,0	4,3	

	1/6 sextile	5/6 sextile	Intersextile range	Skewness	Stnd. skewness	Kurtosis	Stnd. kurtosis	
CR	62,7	68,2	5,5	0,816165	0,999594	0,761229	0,466156	
Cu	61,545	68,05	6,505	0,703989	0,703989	-0,578596	-0,289298	
Total	61,9	68,2	6,3	0,666154	1,05328	0,00807016	0,00638002	

	Sum	Sum of squares	
CR	592,23	39048,0	
Cu	387,09	25020,0	
Total	979,32	64068,0	

The StatAdvisor
This table shows various statistics for each of the 2 columns of data.  To test for significant differences amongst the column means, select Analysis of Variance from the list of Tabular Options.  Select Means Plot from the list of Graphical Options to display the means graphically.  


ANOVA Table
Source	Sum of Squares	Df	Mean Square	F-Ratio	P-Value	
Between groups	5,97529	1	5,97529	0,63	0,4431	
Within groups	124,157	13	9,55055			
Total (Corr.)	130,132	14				

The StatAdvisor
The ANOVA table decomposes the variance of the data into two components: a between-group component and a within-group component.  The F-ratio, which in this case equals 0,625649, is a ratio of the between-group estimate to the within-group estimate.  Since the P-value of the F-test is greater than or equal to 0,05, there is not a statistically significant difference between the means of the 2 variables at the 95,0% confidence level.

Table of Means with 95,0 percent LSD intervals
			Stnd. error			
	Count	Mean	(pooled s)	Lower limit	Upper limit	
CR	9	65,8033	1,03013	64,2297	67,377	
Multiple Range Tests

Method: 95,0 percent LSD
	Count	Mean	Homogeneous Groups	
Cu	6	64,515	X	
CR	9	65,8033	X	

Contrast	Sig.	Difference	+/- Limits	
CR - Cu		1,28833	3,51878	
* denotes a statistically significant difference.

The StatAdvisor
This table applies a multiple comparison procedure to determine which means are significantly different from which others.  The bottom half of the output shows the estimated difference between each pair of means.  There are no statistically significant differences between any pair of means at the 95,0% confidence level.  At the top of the page, one homogenous group is identified by a column of X's.  Within each column, the levels containing X's form a group of means within which there are no statistically significant differences.  The method currently being used to discriminate among the means is Fisher's least significant difference (LSD) procedure.  With this method, there is a 5,0% risk of calling each pair of means significantly different when the actual difference equals 0.  
