# Supplementary material for: Iron and copper on Botrytis cinerea: new inputs in the cellular characterization of their inhibitory effect
Source: PeerJ. 2023 Sep 20;11:e15994. doi: 10.7717/peerj.15994 (PMC10517660; doi:10.7717/peerj.15994)
Supplement: Supplemental Information 1 [file peerj-11-15994-s001.zip › Raw data/Germination Statistics analysis.rtf]

Multiple-Sample Comparison
Sample 1: Cu
Sample 2: Cufe (0)

Sample 1: 4 values ranging from 4,7619 to 8,69565
Sample 2: 3 values ranging from 4,54545 to 7,69231

The StatAdvisor
This procedure compares the data in 2 columns of the current data file.  It constructs various statistical tests and graphs to compare the samples.  The F-test in the ANOVA table will test whether there are any significant differences amongst the means.  If there are, the Multiple Range Tests will tell you which means are significantly different from which others.  If you are worried about the presence of outliers, choose the Kruskal-Wallis Test which compares medians instead of means.  The various plots will help you judge the practical significance of the results, as well as allow you to look for possible violations of the assumptions underlying the analysis of variance.  


Summary Statistics
	Count	Average	Median	Mode	Geometric mean	5% Trimmed mean	5% Winsorized mean	
Cu	4	6,35546	5,98214		6,20128	6,31398	6,35546	
Cufe	3	6,30148	6,66667		6,15433	6,32176	6,30148	
Total	7	6,33232	6,25		6,18111	6,3003	6,33232	

	Variance	Standard deviation	Coeff. of variation	Standard error	5% Winsorized sigma	MAD	
Cu	2,81271	1,67711	26,3886%	0,838557	1,67711	0,744048	
Cufe	2,57569	1,6049	25,4686%	0,926588	1,6049	1,02564	
Total	2,26575	1,50524	23,7708%	0,568928	1,50524	1,44231	

	Sbi	Minimum	Maximum	Range	Lower quartile	Upper quartile	Interquartile range	
Cu	1,52839	4,7619	8,69565	3,93375	5,2381	7,47283	2,23473	
Cufe	1,41932	4,54545	7,69231	3,14685	4,54545	7,69231	3,14685	
Total	1,42951	4,54545	8,69565	4,1502	4,7619	7,69231	2,9304	

	1/6 sextile	5/6 sextile	Intersextile range	Skewness	Stnd. skewness	Kurtosis	Stnd. kurtosis	
Cu	4,7619	8,69565	3,93375	1,20191	0,981358	1,92498	0,785872	
Cufe	4,54545	7,69231	3,14685	-0,970945	-0,686562			
Total	4,7619	7,69231	2,9304	0,406236	0,438785	-0,759196	-0,410013	

	Sum	Sum of squares	
Cu	25,4218	170,006	
Cufe	18,9044	124,277	
Total	44,3263	294,283	

The StatAdvisor
This table shows various statistics for each of the 2 columns of data.  To test for significant differences amongst the column means, select Analysis of Variance from the list of Tabular Options.  Select Means Plot from the list of Graphical Options to display the means graphically.  


ANOVA Table
Source	Sum of Squares	Df	Mean Square	F-Ratio	P-Value	
Between groups	0,00499596	1	0,00499596	0,00	0,9675	
Within groups	13,5895	5	2,71791			
Total (Corr.)	13,5945	6				

The StatAdvisor
The ANOVA table decomposes the variance of the data into two components: a between-group component and a within-group component.  The F-ratio, which in this case equals 0,00183817, is a ratio of the between-group estimate to the within-group estimate.  Since the P-value of the F-test is greater than or equal to 0,05, there is not a statistically significant difference between the means of the 2 variables at the 95,0% confidence level.

Table of Means with 95,0 percent LSD intervals
			Stnd. error			
	Count	Mean	(pooled s)	Lower limit	Upper limit	
Cu	4	6,35546	0,824304	4,85714	7,85378	
Cufe	3	6,30148	0,951824	4,57136	8,03159	
Total	7	6,33232				

The StatAdvisor
This table shows the mean for each column of data.  It also shows the standard error of each mean, which is a measure of its sampling variability.  The standard error is formed by dividing the pooled standard deviation by the square root of the number of observations at each level.  The table also displays an interval around each mean.  The intervals currently displayed are based on Fisher's least significant difference (LSD) procedure.  They are constructed in such a way that if two means are the same, their intervals will overlap 95,0% of the time.  You can display the intervals graphically by selecting Means Plot from the list of Graphical Options.  In the Multiple Range Tests, these intervals are used to determine which means are significantly different from which others.

Multiple Range Tests

Method: 95,0 percent LSD
	Count	Mean	Homogeneous Groups	
Cufe	3	6,30148	X	
Cu	4	6,35546	X	

Contrast	Sig.	Difference	+/- Limits	
Cu - Cufe		0,0539844	3,23674	
* denotes a statistically significant difference.

The StatAdvisor
This table applies a multiple comparison procedure to determine which means are significantly different from which others.  The bottom half of the output shows the estimated difference between each pair of means.  There are no statistically significant differences between any pair of means at the 95,0% confidence level.  At the top of the page, one homogenous group is identified by a column of X's.  Within each column, the levels containing X's form a group of means within which there are no statistically significant differences.  The method currently being used to discriminate among the means is Fisher's least significant difference (LSD) procedure.  With this method, there is a 5,0% risk of calling each pair of means significantly different when the actual difference equals 0.  

Multiple-Sample Comparison
Sample 1: Fe
Sample 2: Cu

Sample 1: 3 values ranging from 4,16667 to 7,40741
Sample 2: 4 values ranging from 4,7619 to 8,69565

The StatAdvisor
This procedure compares the data in 2 columns of the current data file.  It constructs various statistical tests and graphs to compare the samples.  The F-test in the ANOVA table will test whether there are any significant differences amongst the means.  If there are, the Multiple Range Tests will tell you which means are significantly different from which others.  If you are worried about the presence of outliers, choose the Kruskal-Wallis Test which compares medians instead of means.  The various plots will help you judge the practical significance of the results, as well as allow you to look for possible violations of the assumptions underlying the analysis of variance.  


Summary Statistics
	Count	Average	Median	Mode	Geometric mean	5% Trimmed mean	5% Winsorized mean	
Fe	3	5,61241	5,26316		5,45633	5,59301	5,61241	
Cu	4	6,35546	5,98214		6,20128	6,31398	6,35546	
Total	7	6,03701	5,71429		5,87031	5,99322	6,03701	

	Variance	Standard deviation	Coeff. of variation	Standard error	5% Winsorized sigma	MAD	
Fe	2,71708	1,64836	29,3699%	0,95168	1,64836	1,09649	
Cu	2,81271	1,67711	26,3886%	0,838557	1,67711	0,744048	
Total	2,4698	1,57156	26,0321%	0,593994	1,57156	0,952381	

	Sbi	Minimum	Maximum	Range	Lower quartile	Upper quartile	Interquartile range	
Fe	1,4492	4,16667	7,40741	3,24074	4,16667	7,40741	3,24074	
Cu	1,52839	4,7619	8,69565	3,93375	5,2381	7,47283	2,23473	
Total	1,52182	4,16667	8,69565	4,52899	4,7619	7,40741	2,6455	

	1/6 sextile	5/6 sextile	Intersextile range	Skewness	Stnd. skewness	Kurtosis	Stnd. kurtosis	
Fe	4,16667	7,40741	3,24074	0,910653	0,643929			
Cu	4,7619	8,69565	3,93375	1,20191	0,981358	1,92498	0,785872	
Total	4,7619	7,40741	2,6455	0,737161	0,796225	-0,141046	-0,0761733	

	Sum	Sum of squares	
Fe	16,8372	99,9316	
Cu	25,4218	170,006	
Total	42,2591	269,937	

The StatAdvisor
This table shows various statistics for each of the 2 columns of data.  To test for significant differences amongst the column means, select Analysis of Variance from the list of Tabular Options.  Select Means Plot from the list of Graphical Options to display the means graphically.  


ANOVA Table
Source	Sum of Squares	Df	Mean Square	F-Ratio	P-Value	
Between groups	0,946497	1	0,946497	0,34	0,5845	
Within groups	13,8723	5	2,77446			
Total (Corr.)	14,8188	6				

The StatAdvisor
The ANOVA table decomposes the variance of the data into two components: a between-group component and a within-group component.  The F-ratio, which in this case equals 0,341146, is a ratio of the between-group estimate to the within-group estimate.  Since the P-value of the F-test is greater than or equal to 0,05, there is not a statistically significant difference between the means of the 2 variables at the 95,0% confidence level.

Multiple Range Tests

Method: 95,0 percent LSD
	Count	Mean	Homogeneous Groups	
Fe	3	5,61241	X	
Cu	4	6,35546	X	

Contrast	Sig.	Difference	+/- Limits	
Fe - Cu		-0,74305	3,27025	
* denotes a statistically significant difference.

The StatAdvisor
This table applies a multiple comparison procedure to determine which means are significantly different from which others.  The bottom half of the output shows the estimated difference between each pair of means.  There are no statistically significant differences between any pair of means at the 95,0% confidence level.  At the top of the page, one homogenous group is identified by a column of X's.  Within each column, the levels containing X's form a group of means within which there are no statistically significant differences.  The method currently being used to discriminate among the means is Fisher's least significant difference (LSD) procedure.  With this method, there is a 5,0% risk of calling each pair of means significantly different when the actual difference equals 0.  

Multiple-Sample Comparison
Sample 1: Cufe (0)
Sample 2: Fe

Sample 1: 3 values ranging from 4,54545 to 7,69231
Sample 2: 3 values ranging from 4,16667 to 7,40741

The StatAdvisor
This procedure compares the data in 2 columns of the current data file.  It constructs various statistical tests and graphs to compare the samples.  The F-test in the ANOVA table will test whether there are any significant differences amongst the means.  If there are, the Multiple Range Tests will tell you which means are significantly different from which others.  If you are worried about the presence of outliers, choose the Kruskal-Wallis Test which compares medians instead of means.  The various plots will help you judge the practical significance of the results, as well as allow you to look for possible violations of the assumptions underlying the analysis of variance.  


Summary Statistics
	Count	Average	Median	Mode	Geometric mean	5% Trimmed mean	5% Winsorized mean	
Cufe	3	6,30148	6,66667		6,15433	6,32176	6,30148	
Fe	3	5,61241	5,26316		5,45633	5,59301	5,61241	
Total	6	5,95694	5,96491		5,79483	5,95999	5,95694	

	Variance	Standard deviation	Coeff. of variation	Standard error	5% Winsorized sigma	MAD	
Cufe	2,57569	1,6049	25,4686%	0,926588	1,6049	1,02564	
Fe	2,71708	1,64836	29,3699%	0,95168	1,64836	1,09649	
Total	2,25955	1,50318	25,2341%	0,613671	1,50318	1,43098	

	Sbi	Minimum	Maximum	Range	Lower quartile	Upper quartile	Interquartile range	
Cufe	1,41932	4,54545	7,69231	3,14685	4,54545	7,69231	3,14685	
Fe	1,4492	4,16667	7,40741	3,24074	4,16667	7,40741	3,24074	
Total	1,42729	4,16667	7,69231	3,52564	4,54545	7,40741	2,86195	

	1/6 sextile	5/6 sextile	Intersextile range	Skewness	Stnd. skewness	Kurtosis	Stnd. kurtosis	
Cufe	4,54545	7,69231	3,14685	-0,970945	-0,686562			
Fe	4,16667	7,40741	3,24074	0,910653	0,643929			
Total	4,35606	7,54986	3,1938	-0,0219915	-0,0219915	-2,38056	-1,19028	

	Sum	Sum of squares	
Cufe	18,9044	124,277	
Fe	16,8372	99,9316	
Total	35,7417	224,209	

The StatAdvisor
This table shows various statistics for each of the 2 columns of data.  To test for significant differences amongst the column means, select Analysis of Variance from the list of Tabular Options.  Select Means Plot from the list of Graphical Options to display the means graphically.  


ANOVA Table
Source	Sum of Squares	Df	Mean Square	F-Ratio	P-Value	
Between groups	0,712217	1	0,712217	0,27	0,6313	
Within groups	10,5856	4	2,64639			
Total (Corr.)	11,2978	5				

The StatAdvisor
The ANOVA table decomposes the variance of the data into two components: a between-group component and a within-group component.  The F-ratio, which in this case equals 0,269128, is a ratio of the between-group estimate to the within-group estimate.  Since the P-value of the F-test is greater than or equal to 0,05, there is not a statistically significant difference between the means of the 2 variables at the 95,0% confidence level.

Table of Means with 95,0 percent LSD intervals
			Stnd. error			
	Count	Mean	(pooled s)	Lower limit	Upper limit	
Cufe	3	6,30148	0,939218	4,45756	8,14539	
Fe	3	5,61241	0,939218	3,76849	7,45633	
Total	6	5,95694				

The StatAdvisor
This table shows the mean for each column of data.  It also shows the standard error of each mean, which is a measure of its sampling variability.  The standard error is formed by dividing the pooled standard deviation by the square root of the number of observations at each level.  The table also displays an interval around each mean.  The intervals currently displayed are based on Fisher's least significant difference (LSD) procedure.  They are constructed in such a way that if two means are the same, their intervals will overlap 95,0% of the time.  You can display the intervals graphically by selecting Means Plot from the list of Graphical Options.  In the Multiple Range Tests, these intervals are used to determine which means are significantly different from which others.

Multiple-Sample Comparison
Sample 1: Cu
Sample 2: Cufe (0)

Sample 1: 4 values ranging from 4,7619 to 8,69565
Sample 2: 3 values ranging from 4,54545 to 7,69231

The StatAdvisor
This procedure compares the data in 2 columns of the current data file.  It constructs various statistical tests and graphs to compare the samples.  The F-test in the ANOVA table will test whether there are any significant differences amongst the means.  If there are, the Multiple Range Tests will tell you which means are significantly different from which others.  If you are worried about the presence of outliers, choose the Kruskal-Wallis Test which compares medians instead of means.  The various plots will help you judge the practical significance of the results, as well as allow you to look for possible violations of the assumptions underlying the analysis of variance.  


Summary Statistics
	Count	Average	Median	Mode	Geometric mean	5% Trimmed mean	5% Winsorized mean	
Cu	4	6,35546	5,98214		6,20128	6,31398	6,35546	
Cufe	3	6,30148	6,66667		6,15433	6,32176	6,30148	
Total	7	6,33232	6,25		6,18111	6,3003	6,33232	

	Variance	Standard deviation	Coeff. of variation	Standard error	5% Winsorized sigma	MAD	
Cu	2,81271	1,67711	26,3886%	0,838557	1,67711	0,744048	
Cufe	2,57569	1,6049	25,4686%	0,926588	1,6049	1,02564	
Total	2,26575	1,50524	23,7708%	0,568928	1,50524	1,44231	

	Sbi	Minimum	Maximum	Range	Lower quartile	Upper quartile	Interquartile range	
Cu	1,52839	4,7619	8,69565	3,93375	5,2381	7,47283	2,23473	
Cufe	1,41932	4,54545	7,69231	3,14685	4,54545	7,69231	3,14685	
Total	1,42951	4,54545	8,69565	4,1502	4,7619	7,69231	2,9304	

	1/6 sextile	5/6 sextile	Intersextile range	Skewness	Stnd. skewness	Kurtosis	Stnd. kurtosis	
Cu	4,7619	8,69565	3,93375	1,20191	0,981358	1,92498	0,785872	
Cufe	4,54545	7,69231	3,14685	-0,970945	-0,686562			
Total	4,7619	7,69231	2,9304	0,406236	0,438785	-0,759196	-0,410013	

	Sum	Sum of squares	
Cu	25,4218	170,006	
Cufe	18,9044	124,277	
Total	44,3263	294,283	

The StatAdvisor
This table shows various statistics for each of the 2 columns of data.  To test for significant differences amongst the column means, select Analysis of Variance from the list of Tabular Options.  Select Means Plot from the list of Graphical Options to display the means graphically.  


ANOVA Table
Source	Sum of Squares	Df	Mean Square	F-Ratio	P-Value	
Between groups	0,00499596	1	0,00499596	0,00	0,9675	
Within groups	13,5895	5	2,71791			
Total (Corr.)	13,5945	6				

The StatAdvisor
The ANOVA table decomposes the variance of the data into two components: a between-group component and a within-group component.  The F-ratio, which in this case equals 0,00183817, is a ratio of the between-group estimate to the within-group estimate.  Since the P-value of the F-test is greater than or equal to 0,05, there is not a statistically significant difference between the means of the 2 variables at the 95,0% confidence level.

Table of Means with 95,0 percent LSD intervals
			Stnd. error			
	Count	Mean	(pooled s)	Lower limit	Upper limit	
Cu	4	6,35546	0,824304	4,85714	7,85378	
Cufe	3	6,30148	0,951824	4,57136	8,03159	
Total	7	6,33232				

The StatAdvisor
This table shows the mean for each column of data.  It also shows the standard error of each mean, which is a measure of its sampling variability.  The standard error is formed by dividing the pooled standard deviation by the square root of the number of observations at each level.  The table also displays an interval around each mean.  The intervals currently displayed are based on Fisher's least significant difference (LSD) procedure.  They are constructed in such a way that if two means are the same, their intervals will overlap 95,0% of the time.  You can display the intervals graphically by selecting Means Plot from the list of Graphical Options.  In the Multiple Range Tests, these intervals are used to determine which means are significantly different from which others.

Multiple Range Tests

Method: 95,0 percent LSD
	Count	Mean	Homogeneous Groups	
Cufe	3	6,30148	X	
Cu	4	6,35546	X	

Contrast	Sig.	Difference	+/- Limits	
Cu - Cufe		0,0539844	3,23674	
* denotes a statistically significant difference.

The StatAdvisor
This table applies a multiple comparison procedure to determine which means are significantly different from which others.  The bottom half of the output shows the estimated difference between each pair of means.  There are no statistically significant differences between any pair of means at the 95,0% confidence level.  At the top of the page, one homogenous group is identified by a column of X's.  Within each column, the levels containing X's form a group of means within which there are no statistically significant differences.  The method currently being used to discriminate among the means is Fisher's least significant difference (LSD) procedure.  With this method, there is a 5,0% risk of calling each pair of means significantly different when the actual difference equals 0.  
