# Supplementary material for: Iron and copper on Botrytis cinerea: new inputs in the cellular characterization of their inhibitory effect
Source: PeerJ. 2023 Sep 20;11:e15994. doi: 10.7717/peerj.15994 (PMC10517660; doi:10.7717/peerj.15994)
Supplement: Supplemental Information 1 [file peerj-11-15994-s001.zip › Raw data/Wild strain analysis Statistics analysis.rtf]

Multiple-Sample Comparison
Sample 1: CuFe (c-1912) Bcpo03
Sample 2: Fe 1912

Sample 1: 3 values ranging from 5,66 to 5,88
Sample 2: 3 values ranging from 5,95 to 7,03

The StatAdvisor
This procedure compares the data in 2 columns of the current data file.  It constructs various statistical tests and graphs to compare the samples.  The F-test in the ANOVA table will test whether there are any significant differences amongst the means.  If there are, the Multiple Range Tests will tell you which means are significantly different from which others.  If you are worried about the presence of outliers, choose the Kruskal-Wallis Test which compares medians instead of means.  The various plots will help you judge the practical significance of the results, as well as allow you to look for possible violations of the assumptions underlying the analysis of variance.  


Table of Means with 95,0 percent LSD intervals
			Stnd. error			
	Count	Mean	(pooled s)	Lower limit	Upper limit	
CuFe (c-1912) Bcpo03	3	5,78333	0,250954	5,29065	6,27602	
Fe 1912	3	6,64667	0,250954	6,15398	7,13935	
Total	6	6,215				

The StatAdvisor
This table shows the mean for each column of data.  It also shows the standard error of each mean, which is a measure of its sampling variability.  The standard error is formed by dividing the pooled standard deviation by the square root of the number of observations at each level.  The table also displays an interval around each mean.  The intervals currently displayed are based on Fisher's least significant difference (LSD) procedure.  They are constructed in such a way that if two means are the same, their intervals will overlap 95,0% of the time.  You can display the intervals graphically by selecting Means Plot from the list of Graphical Options.  In the Multiple Range Tests, these intervals are used to determine which means are significantly different from which others.

Multiple-Sample Comparison
Sample 1: CuFe (C-1911) Bcad03
Sample 2: Fe 1911

Sample 1: 3 values ranging from 6,36 to 6,96
Sample 2: 3 values ranging from 5,96 to 6,23

The StatAdvisor
This procedure compares the data in 2 columns of the current data file.  It constructs various statistical tests and graphs to compare the samples.  The F-test in the ANOVA table will test whether there are any significant differences amongst the means.  If there are, the Multiple Range Tests will tell you which means are significantly different from which others.  If you are worried about the presence of outliers, choose the Kruskal-Wallis Test which compares medians instead of means.  The various plots will help you judge the practical significance of the results, as well as allow you to look for possible violations of the assumptions underlying the analysis of variance.  


Summary Statistics
	Count	Average	Median	Mode	Geometric mean	5% Trimmed mean	
CuFe (C-1911) Bcad03	3	6,62	6,54		6,61528	6,61556	
Fe 1911	3	6,07333	6,03		6,07226	6,07093	
Total	6	6,34667	6,295		6,33796	6,33407	

	5% Winsorized mean	Variance	Standard deviation	Coeff. of variation	
CuFe (C-1911) Bcad03	6,62	0,0948	0,307896	4,651%	
Fe 1911	6,07333	0,0196333	0,140119	2,30712%	
Total	6,34667	0,135427	0,368004	5,79838%	

	Standard error	5% Winsorized sigma	MAD	Sbi	Minimum	Maximum	
CuFe (C-1911) Bcad03	0,177764	0,307896	0,18	0,275817	6,36	6,96	
Fe 1911	0,0808977	0,140119	0,07	0,128141	5,96	6,23	
Total	0,150237	0,368004	0,255	0,345001	5,96	6,96	

	Range	Lower quartile	Upper quartile	Interquartile range	1/6 sextile	5/6 sextile	
CuFe (C-1911) Bcad03	0,6	6,36	6,96	0,6	6,36	6,96	
Fe 1911	0,27	5,96	6,23	0,27	5,96	6,23	
Total	1,0	6,03	6,54	0,51	5,995	6,75	

	Intersextile range	Skewness	Stnd. skewness	Kurtosis	Stnd. kurtosis	Sum	
CuFe (C-1911) Bcad03	0,6	1,09029	0,770952			19,86	
Fe 1911	0,27	1,25857	0,889944			18,22	
Total	0,755	0,883657	0,883657	0,448384	0,224192	38,08	

	Sum of squares	
CuFe (C-1911) Bcad03	131,663	
Fe 1911	110,695	
Total	242,358	

The StatAdvisor
This table shows various statistics for each of the 2 columns of data.  To test for significant differences amongst the column means, select Analysis of Variance from the list of Tabular Options.  Select Means Plot from the list of Graphical Options to display the means graphically.  


Table of Means with 95,0 percent LSD intervals
			Stnd. error			
	Count	Mean	(pooled s)	Lower limit	Upper limit	
CuFe (C-1911) Bcad03	3	6,62	0,138102	6,34887	6,89113	
Fe 1911	3	6,07333	0,138102	5,8022	6,34446	
Total	6	6,34667				

The StatAdvisor
This table shows the mean for each column of data.  It also shows the standard error of each mean, which is a measure of its sampling variability.  The standard error is formed by dividing the pooled standard deviation by the square root of the number of observations at each level.  The table also displays an interval around each mean.  The intervals currently displayed are based on Fisher's least significant difference (LSD) procedure.  They are constructed in such a way that if two means are the same, their intervals will overlap 95,0% of the time.  You can display the intervals graphically by selecting Means Plot from the list of Graphical Options.  In the Multiple Range Tests, these intervals are used to determine which means are significantly different from which others.

Multiple Range Tests

Method: 95,0 percent LSD
	Count	Mean	Homogeneous Groups	
Fe 1911	3	6,07333	X	
CuFe (C-1911) Bcad03	3	6,62	 X	

Contrast	Sig.	Difference	+/- Limits	
CuFe (C-1911) Bcad03 - Fe 1911	 *	0,546667	0,542258	
* denotes a statistically significant difference.

The StatAdvisor
This table applies a multiple comparison procedure to determine which means are significantly different from which others.  The bottom half of the output shows the estimated difference between each pair of means.  An asterisk has been placed next to 1 pair, indicating that this pair shows a statistically significant difference at the 95,0% confidence level.  At the top of the page, 2 homogenous groups are identified using columns of X's.  Within each column, the levels containing X's form a group of means within which there are no statistically significant differences.  The method currently being used to discriminate among the means is Fisher's least significant difference (LSD) procedure.  With this method, there is a 5,0% risk of calling each pair of means significantly different when the actual difference equals 0.  


Summary Statistics
	Count	Average	Median	Mode	Geometric mean	5% Trimmed mean	
CuFe 2025 Bcvi09	3	6,44	6,41		6,4394	6,43833	
Fe 2025	3	6,54	6,46		6,52908	6,53556	
Total	6	6,49	6,435		6,48408	6,48	

	5% Winsorized mean	Variance	Standard deviation	Coeff. of variation	
CuFe 2025 Bcvi09	6,44	0,0117	0,108167	1,6796%	
Fe 2025	6,54	0,2164	0,465188	7,11297%	
Total	6,49	0,09424	0,306985	4,73013%	

	Standard error	5% Winsorized sigma	MAD	Sbi	Minimum	Maximum	
CuFe 2025 Bcvi09	0,06245	0,108167	0,06	0,0976051	6,35	6,56	
Fe 2025	0,268576	0,465188	0,34	0,403396	6,12	7,04	
Total	0,125326	0,306985	0,105	0,243446	6,12	7,04	

	Range	Lower quartile	Upper quartile	Interquartile range	1/6 sextile	5/6 sextile	
CuFe 2025 Bcvi09	0,21	6,35	6,56	0,21	6,35	6,56	
Fe 2025	0,92	6,12	7,04	0,92	6,12	7,04	
Total	0,92	6,35	6,56	0,21	6,235	6,8	

	Intersextile range	Skewness	Stnd. skewness	Kurtosis	Stnd. kurtosis	Sum	
CuFe 2025 Bcvi09	0,21	1,15207	0,814636			19,32	
Fe 2025	0,92	0,750993	0,531032			19,62	
Total	0,565	1,16952	1,16952	2,47502	1,23751	38,94	

	Sum of squares	
CuFe 2025 Bcvi09	124,444	
Fe 2025	128,748	
Total	253,192	

The StatAdvisor
This table shows various statistics for each of the 2 columns of data.  To test for significant differences amongst the column means, select Analysis of Variance from the list of Tabular Options.  Select Means Plot from the list of Graphical Options to display the means graphically.  

WARNING: There is more than a 3 to 1 difference between the smallest standard deviation and the largest.  This may cause problems since the analysis of variance assumes that the standard deviations at all levels are equal.  Select Variance Check from the list of Tabular Options to run a formal statistical test for differences among the sigmas.  You may want to consider transforming the data to remove any dependence of the standard deviation on the mean.  


Table of Means with 95,0 percent LSD intervals
			Stnd. error			
	Count	Mean	(pooled s)	Lower limit	Upper limit	
CuFe 2025 Bcvi09	3	6,44	0,194979	6,05721	6,82279	
Fe 2025	3	6,54	0,194979	6,15721	6,92279	
Total	6	6,49				

The StatAdvisor
This table shows the mean for each column of data.  It also shows the standard error of each mean, which is a measure of its sampling variability.  The standard error is formed by dividing the pooled standard deviation by the square root of the number of observations at each level.  The table also displays an interval around each mean.  The intervals currently displayed are based on Fisher's least significant difference (LSD) procedure.  They are constructed in such a way that if two means are the same, their intervals will overlap 95,0% of the time.  You can display the intervals graphically by selecting Means Plot from the list of Graphical Options.  In the Multiple Range Tests, these intervals are used to determine which means are significantly different from which others.

Multiple Range Tests

Method: 95,0 percent LSD
	Count	Mean	Homogeneous Groups	
CuFe 2025 Bcvi09	3	6,44	X	
Fe 2025	3	6,54	X	

Contrast	Sig.	Difference	+/- Limits	
CuFe 2025 Bcvi09 - Fe 2025		-0,1	0,765583	
* denotes a statistically significant difference.

The StatAdvisor
This table applies a multiple comparison procedure to determine which means are significantly different from which others.  The bottom half of the output shows the estimated difference between each pair of means.  There are no statistically significant differences between any pair of means at the 95,0% confidence level.  At the top of the page, one homogenous group is identified by a column of X's.  Within each column, the levels containing X's form a group of means within which there are no statistically significant differences.  The method currently being used to discriminate among the means is Fisher's least significant difference (LSD) procedure.  With this method, there is a 5,0% risk of calling each pair of means significantly different when the actual difference equals 0.  
