# Supplementary material for: Iron and copper on Botrytis cinerea: new inputs in the cellular characterization of their inhibitory effect
Source: PeerJ. 2023 Sep 20;11:e15994. doi: 10.7717/peerj.15994 (PMC10517660; doi:10.7717/peerj.15994)
Supplement: Supplemental Information 2 [file peerj-11-15994-s002.docx]

LOCUS OP852422 290 bp DNA linear PLN 17-NOV-2022

DEFINITION Botrytis cinerea isolate Bc.ad03 internal transcribed spacer 1,

partial sequence; 5.8S ribosomal RNA gene, complete sequence; and

internal transcribed spacer 2, partial sequence.

ACCESSION OP852422

VERSION OP852422

KEYWORDS .

SOURCE Botrytis cinerea (Botryotinia fuckeliana)

ORGANISM Botrytis cinerea

Eukaryota; Fungi; Dikarya; Ascomycota; Pezizomycotina;

Leotiomycetes; Helotiales; Sclerotiniaceae; Botrytis.

REFERENCE 1 (bases 1 to 290)

AUTHORS Castillo,L., Aguilera,L., Plaza,V., Olivares,L. and Marambio,B.

TITLE ITS B cinerea 3 aislados

JOURNAL Unpublished

REFERENCE 2 (bases 1 to 290)

AUTHORS Castillo,L., Aguilera,L., Plaza,V., Olivares,L. and Marambio,B.

TITLE Direct Submission

JOURNAL Submitted (17-NOV-2022) Departamento de Biologia, Universidad de La

Serena, Raul Bitran 1305, La Serena 1720256, Chile

COMMENT ##Assembly-Data-START##

Sequencing Technology :: Sanger dideoxy sequencing

##Assembly-Data-END##

FEATURES Location/Qualifiers

source 1..290

/organism="Botrytis cinerea"

/mol_type="genomic DNA"

/isolate="Bc.ad03"

/db_xref="taxon:40559"

misc_RNA <1..>290

/note="contains internal transcribed spacer 1, 5.8S

ribosomal RNA, and internal transcribed spacer 2"

ORIGIN

1 ccgggccttg taggctggcc agagaatacc aaaactctct ttattaatgt cgtctgagta

61 ctatataata gttaaaactt tcaacaacgg atctcttggt tctggcatcg atgaagaacg

121 cagcgaaatg cgataagtaa tgtgaattgc agaattcagt gaatcatcga atctttgaac

181 gcacattgcg ccccttggta ttccgggggg catgcctgtt cgagcgtcat ttcaaccctc

241 aagcttagct tggtatggag tctatgtcag taatggcagg ctgtaaaatc

//

LOCUS OP852423 506 bp DNA linear PLN 17-NOV-2022

DEFINITION Botrytis cinerea isolate Bc.po03 small subunit ribosomal RNA gene,

partial sequence; internal transcribed spacer 1 and 5.8S ribosomal

RNA gene, complete sequence; and internal transcribed spacer 2,

partial sequence.

ACCESSION OP852423

VERSION OP852423

KEYWORDS .

SOURCE Botrytis cinerea (Botryotinia fuckeliana)

ORGANISM Botrytis cinerea

Eukaryota; Fungi; Dikarya; Ascomycota; Pezizomycotina;

Leotiomycetes; Helotiales; Sclerotiniaceae; Botrytis.

REFERENCE 1 (bases 1 to 506)

AUTHORS Castillo,L., Aguilera,L., Plaza,V., Olivares,L. and Marambio,B.

TITLE ITS B cinerea 3 aislados

JOURNAL Unpublished

REFERENCE 2 (bases 1 to 506)

AUTHORS Castillo,L., Aguilera,L., Plaza,V., Olivares,L. and Marambio,B.

TITLE Direct Submission

JOURNAL Submitted (17-NOV-2022) Departamento de Biologia, Universidad de La

Serena, Raul Bitran 1305, La Serena 1720256, Chile

COMMENT ##Assembly-Data-START##

Sequencing Technology :: Sanger dideoxy sequencing

##Assembly-Data-END##

FEATURES Location/Qualifiers

source 1..506

/organism="Botrytis cinerea"

/mol_type="genomic DNA"

/isolate="Bc.po03"

/db_xref="taxon:40559"

misc_RNA <1..>506

/note="contains small subunit ribosomal RNA, internal

transcribed spacer 1, 5.8S ribosomal RNA, and internal

transcribed spacer 2"

ORIGIN

1 cttggtcatt tagaggaagt aaaagtcgta acaaggtttc cgtaggtgaa cctgcggaag

61 gatcattaca gagttcatgc ccgaaagggt agacctccca cccttgtgta ttattacttt

121 gttgctttgg cgagctgcct tcgggccttg tatgctcgcc agagaatacc aaaactcttt

181 ttattaatgt cgtctgagta ctatataata gttaaaactt tcaacaacgg atctcttggt

241 tctggcatcg atgaagaacg cagcgaaatg cgataagtaa tgtgaattgc agaattcagt

301 gaatcatcga atctttgaac gcacattgcg ccccttggta ttccgggggg catgcctgtt

361 cgagcgtcat ttcaaccctc aagcttagct tggtattgag tctatgtcag taatggcagg

421 ctctaaaatc agtggcggcg ccgctgggtc ctgaacgtag taatatctct cgttacaggt

481 tcttggtgtt cttctgccaa aaccca

//

LOCUS OP852424 538 bp DNA linear PLN 17-NOV-2022

DEFINITION Botrytis cinerea isolate Bc.vi09 small subunit ribosomal RNA gene,

partial sequence; internal transcribed spacer 1 and 5.8S ribosomal

RNA gene, complete sequence; and internal transcribed spacer 2,

partial sequence.

ACCESSION OP852424

VERSION OP852424

KEYWORDS .

SOURCE Botrytis cinerea (Botryotinia fuckeliana)

ORGANISM Botrytis cinerea

Eukaryota; Fungi; Dikarya; Ascomycota; Pezizomycotina;

Leotiomycetes; Helotiales; Sclerotiniaceae; Botrytis.

REFERENCE 1 (bases 1 to 538)

AUTHORS Castillo,L., Aguilera,L., Plaza,V., Olivares,L. and Marambio,B.

TITLE ITS B cinerea 3 aislados

JOURNAL Unpublished

REFERENCE 2 (bases 1 to 538)

AUTHORS Castillo,L., Aguilera,L., Plaza,V., Olivares,L. and Marambio,B.

TITLE Direct Submission

JOURNAL Submitted (17-NOV-2022) Departamento de Biologia, Universidad de La

Serena, Raul Bitran 1305, La Serena 1720256, Chile

COMMENT ##Assembly-Data-START##

Sequencing Technology :: Sanger dideoxy sequencing

##Assembly-Data-END##

FEATURES Location/Qualifiers

source 1..538

/organism="Botrytis cinerea"

/mol_type="genomic DNA"

/isolate="Bc.vi09"

/db_xref="taxon:40559"

misc_RNA <1..>538

/note="contains small subunit ribosomal RNA, internal

transcribed spacer 1, 5.8S ribosomal RNA, and internal

transcribed spacer 2"

ORIGIN

1 tcttggttcc attagaggaa gtaaaagtcg taacaaggtt tccgtaggtg aacctgcgga

61 aggatcatta cagagttcat gcccgaaagg gtagacctcc cacccttgtg tattattact

121 ttgttgcttt ggcgagctgc cttcgggcct tgtatgctcg ccagagaata ccaaaactct

181 ttttattaat gtcgtctgag tactatataa tagttaaaac tttcaacaac ggatctcttg

241 gttctggcat cgatgaagaa cgcagcgaaa tgcgataagt aatgtgaatt gcagaattca

301 gtgaatcatc gaatctttga acgcacattg cgccccttgg tattccgggg ggcatgcctg

361 ttcgagcgtc atttcaaccc tcaagcttag cttggtattg agtctatgtc agtaatggca

421 ggctctaaaa tcagtggcgg cgccgctggg tcctgaacgt agtaatatct ctcgttacag

481 gttctcggtg tgcttctgcc aaaacccaaa tttttctatg gtgacctcgg atcaggta

//

LOCUS Seq1 926 bp DNA linear PLN 17-NOV-2022

DEFINITION Botrytis cinerea Bc.ad03 RPB2 DNA, parcial CDS.

ACCESSION Seq1

VERSION

KEYWORDS .

SOURCE Botrytis cinerea (Botryotinia fuckeliana)

ORGANISM Botrytis cinerea

Eukaryota; Fungi; Dikarya; Ascomycota; Pezizomycotina;

Leotiomycetes; Helotiales; Sclerotiniaceae; Botrytis.

REFERENCE 1 (bases 1 to 926)

AUTHORS Castillo,L., Aguilera,L., Plaza,V., Olivares,L. and Marambio,B.

TITLE Direct Submission

JOURNAL Submitted (17-NOV-2022) Departamento de Biologia, Universidad de La

Serena, Raul Bitran 1305, La Serena, 1720256, Chile

COMMENT Bankit Comment: TOTAL # OF SEQS:3

##Assembly-Data-START##

Sequencing Technology :: Sanger dideoxy sequencing

##Assembly-Data-END##

FEATURES Location/Qualifiers

source 1..926

/organism="Botrytis cinerea"

/mol_type="genomic DNA"

/db_xref="taxon:40559"

gene <1..>926

/gene="RPB2"

CDS <2..925

/gene="RPB2"

/codon_start=1

/product="RNA polymerase II second largest subunit"

/translation="LTTDVYRYLQRCVENNREFNLTLGVKSTTITNGLKYSLATGNWG

DQKKAASSTAGVSQVLNRYTFASTLSHLRRTNTPIGRDGKIAKPRQLHNTHWGLVCPA

ETPEGQACGLVKNLALMCYVTVGTPSDPIVEFMIQRNMEVLEEYEPLRAPNATKVFVN

GVWVGIHRDPAHLVKCVQDLRRSHLISHEVSLIREIRDREFKIFTDAGRVCRPLLVID

NDPDSANKGNLVLNKDHIRRLEDDQLLPANMDKDEKVRNGYYGFQGLINDGVVEYLDA

EEEETVMITMTPEDLDISRQLQAGYQIRPDES"

BASE COUNT 271 a 189 c 227 g 239 t

ORIGIN

1 attgacaaca gacgtgtaca gatacttgca acgttgcgtg gaaaacaacc gagagtttaa

61 tttgactttg ggtgtgaaat caacaacaat caccaacggt ctgaaatatt ctttggccac

121 aggtaactgg ggtgaccaga agaaggcagc aagttctacc gccggagtgt ctcaagtgtt

181 gaacagatat acctttgcat ccacactttc tcatttgcgc cgaaccaata cacccatcgg

241 acgtgatgga aagatcgcca aacctagaca gctgcataat acccattggg gcttggtctg

301 tccggcagag acgcccgaag gacaagcttg tggtttggtt aagaatttgg ctctgatgtg

361 ttacgttaca gtcggtacgc caagtgatcc aatcgttgag ttcatgattc aacgaaatat

421 ggaagtattg gaggagtatg aaccactccg agcccccaat gcaacaaagg ttttcgtcaa

481 tggtgtttgg gttggtattc atcgagatcc tgctcatttg gtcaaatgtg tccaagatct

541 tcgtagatca cacttgatct ctcatgaagt ttcacttatt cgagaaattc gtgatagaga

601 attcaagatt ttcacagatg caggacgagt gtgcagacct ctattggtta ttgacaatga

661 tcctgacagc gcaaacaaag gtaacttggt gttgaacaag gatcacattc gccgtctgga

721 ggatgatcaa ttgctaccag caaacatgga taaggatgag aaagtaagaa acggatacta

781 tggattccaa ggtttgatta atgacggtgt ggttgagtac ctggatgccg aggaagaaga

841 gactgtcatg attacaatga cacctgaaga tctggacatc tcccgacagc ttcaggctgg

901 ttaccaaatt cgtcctgacg aaagtg

//

LOCUS Seq2 926 bp DNA linear PLN 17-NOV-2022

DEFINITION Botrytis cinerea Bc.po03 RPB2 DNA, parcial CDS.

ACCESSION Seq2

VERSION

KEYWORDS .

SOURCE Botrytis cinerea (Botryotinia fuckeliana)

ORGANISM Botrytis cinerea

Eukaryota; Fungi; Dikarya; Ascomycota; Pezizomycotina;

Leotiomycetes; Helotiales; Sclerotiniaceae; Botrytis.

REFERENCE 1 (bases 1 to 926)

AUTHORS Castillo,L., Aguilera,L., Plaza,V., Olivares,L. and Marambio,B.

TITLE Direct Submission

JOURNAL Submitted (17-NOV-2022) Departamento de Biologia, Universidad de La

Serena, Raul Bitran 1305, La Serena, 1720256, Chile

COMMENT Bankit Comment: TOTAL # OF SEQS:3

##Assembly-Data-START##

Sequencing Technology :: Sanger dideoxy sequencing

##Assembly-Data-END##

FEATURES Location/Qualifiers

source 1..926

/organism="Botrytis cinerea"

/mol_type="genomic DNA"

/db_xref="taxon:40559"

gene <1..>926

/gene="RPB2"

CDS <2..925

/gene="RPB2"

/codon_start=1

/product="RNA polymerase II second largest subunit"

/translation="LTTDVYRYLQRCVENNREFNLTLGVKSTTITNGLKYSLATGNWG

DQKKAASSTAGVSQVLNRYTFASTLSHLRRTNTPIGRDGKIAKPRQLHNTHWGLVCPA

ETPEGQACGLVKNLALMCYVTVGTPSDPIVEFMIQRNMEVLEEYEPLRAPNATKVFVN

GVWVGIHRDPAHLVKCVQDLRRSHLISHEVSLIREIRDREFKIFTDAGRVCRPLLVID

NDPDSANKGNLVLNKDHIRRLEDDQLLPANMDKDEKVRNGYYGFQGLINDGVVEYLDA

EEEETVMITMTPEDLDISRQLQAGYQIRPDES"

BASE COUNT 271 a 189 c 227 g 239 t

ORIGIN

1 attgacaaca gacgtgtaca gatacttgca acgttgcgtg gaaaacaacc gagagtttaa

61 tttgactttg ggtgtgaaat caacaacaat caccaacggt ctgaaatatt ctttggccac

121 aggtaactgg ggtgaccaga agaaggcagc aagttctacc gccggagtgt ctcaagtgtt

181 gaacagatat acctttgcat ccacactttc tcatttgcgc cgaaccaata cacccatcgg

241 acgtgatgga aagatcgcca aacctagaca gctgcataat acccattggg gcttggtctg

301 tccggcagag acgcccgaag gacaagcttg tggtttggtt aagaatttgg ctctgatgtg

361 ttacgttaca gtcggtacgc caagtgatcc aatcgttgag ttcatgattc aacgaaatat

421 ggaagtattg gaggagtatg aaccactccg agcccccaat gcaacaaagg ttttcgtcaa

481 tggtgtttgg gttggtattc atcgagatcc tgctcatttg gtcaaatgtg tccaagatct

541 tcgtagatca cacttgatct ctcatgaagt ttcacttatt cgagaaattc gtgatagaga

601 attcaagatt ttcacagatg caggacgagt gtgcagacct ctattggtta ttgacaatga

661 tcctgacagc gcaaacaaag gtaacttggt gttgaacaag gatcacattc gccgtctgga

721 ggatgatcaa ttgctaccag caaacatgga taaggatgag aaagtaagaa acggatacta

781 tggattccaa ggtttgatta atgacggtgt ggttgagtac ctggatgccg aggaagaaga

841 gactgtcatg attacaatga cacctgaaga tctggacatc tcccgacagc ttcaggctgg

901 ttaccaaatt cgtcctgacg aaagtg

//

LOCUS Seq3 926 bp DNA linear PLN 17-NOV-2022

DEFINITION Botrytis cinerea Bc.vi09 RPB2 DNA, parcial CDS.

ACCESSION Seq3

VERSION

KEYWORDS .

SOURCE Botrytis cinerea (Botryotinia fuckeliana)

ORGANISM Botrytis cinerea

Eukaryota; Fungi; Dikarya; Ascomycota; Pezizomycotina;

Leotiomycetes; Helotiales; Sclerotiniaceae; Botrytis.

REFERENCE 1 (bases 1 to 926)

AUTHORS Castillo,L., Aguilera,L., Plaza,V., Olivares,L. and Marambio,B.

TITLE Direct Submission

JOURNAL Submitted (17-NOV-2022) Departamento de Biologia, Universidad de La

Serena, Raul Bitran 1305, La Serena, 1720256, Chile

COMMENT Bankit Comment: TOTAL # OF SEQS:3

##Assembly-Data-START##

Sequencing Technology :: Sanger dideoxy sequencing

##Assembly-Data-END##

FEATURES Location/Qualifiers

source 1..926

/organism="Botrytis cinerea"

/mol_type="genomic DNA"

/db_xref="taxon:40559"

gene <1..>926

/gene="RPB2"

CDS <2..925

/gene="RPB2"

/codon_start=1

/product="RNA polymerase II second largest subunit"

/translation="LTTDVYRYLQRCVENNREFNLTLGVKSTTITNGLKYSLATGNWG

DQKKAASSTAGVSQVLNRYTFASTLSHLRRTNTPIGRDGKIAKPRQLHNTHWGLVCPA

ETPEGQACGLVKNLALMCYVTVGTPSDPIVEFMIQRNMEVLEEYEPLRAPNATKVFVN

GVWVGIHRDPAHLVKCVQDLRRSHLISHEVSLIREIRDREFKIFTDAGRVCRPLLVID

NDPDSANKGNLVLNKDHIRRLEDDQLLPANMDKDEKVRNGYYGFQGLINDGVVEYLDA

EEEETVMITMTPEDLDISRQLQAGYQIRPDES"

BASE COUNT 270 a 188 c 228 g 240 t

ORIGIN

1 attgacaaca gacgtgtaca gatacttgca acgttgcgtg gaaaacaacc gagagtttaa

61 tttgactttg ggtgtgaaat caacaacaat caccaacggt ctgaaatatt ctttggccac

121 aggtaactgg ggtgaccaga agaaggcagc aagttctacc gccggagtgt ctcaagtgtt

181 gaacagatat acctttgcat ccacactttc tcatttgcgc cgaaccaata cacccattgg

241 acgtgatgga aagatcgcca aacctagaca gctgcataat acccattggg gcttggtctg

301 tccggcagag acgcccgaag gacaagcttg tggtttggtt aagaatttgg ctctgatgtg

361 ttacgttaca gtcggtacgc caagtgatcc aatcgttgag ttcatgattc aacgaaatat

421 ggaagtattg gaggagtatg aaccactccg agcccccaat gcaacaaagg ttttcgtcaa

481 tggtgtttgg gttggtattc atcgagatcc tgctcatttg gtcaaatgtg tccaagatct

541 tcgtagatca cacttgatct ctcatgaagt ttcacttatt cgagaaattc gtgatagaga

601 attcaagatt ttcacagatg caggacgagt gtgcagacct ctattggtta ttgacaatga

661 tcctgacagc gcaaacaaag gtaacttggt gttgaacaag gatcacattc gccgtctgga

721 ggatgatcag ttgctaccag caaacatgga taaggatgag aaagtaagaa acggatacta

781 tggattccaa ggtttgatta atgacggtgt ggttgagtac ctggatgccg aggaagaaga

841 gactgtcatg attacaatga cacctgaaga tctggacatc tcccgacagc ttcaggctgg

901 ttaccaaatt cgtcctgacg aaagtg

//

LOCUS Seq1 705 bp DNA linear PLN 18-NOV-2022

DEFINITION Botrytis cinerea Bc.ad03 HSP60 DNA, parcial CDS.

ACCESSION Seq1

VERSION

KEYWORDS .

SOURCE Botrytis cinerea (Botryotinia fuckeliana)

ORGANISM Botrytis cinerea

Eukaryota; Fungi; Dikarya; Ascomycota; Pezizomycotina;

Leotiomycetes; Helotiales; Sclerotiniaceae; Botrytis.

REFERENCE 1 (bases 1 to 705)

AUTHORS Castillo,L., Aguilera,L., Plaza,V., Olivares,L. and Marambio,B.

TITLE Direct Submission

JOURNAL Submitted (18-NOV-2022) Departamento de Biologia, Universidad de La

Serena, Raul Bitran 1305, La Serena, 1720256, Chile

COMMENT Bankit Comment: TOTAL # OF SEQS:3

##Assembly-Data-START##

Sequencing Technology :: Sanger dideoxy sequencing

##Assembly-Data-END##

FEATURES Location/Qualifiers

source 1..705

/organism="Botrytis cinerea"

/mol_type="genomic DNA"

/db_xref="taxon:40559"

gene <1..>705

/gene="HSP60"

CDS <1..>705

/gene="HSP60"

/codon_start=1

/product="heat shock protein 60"

/translation="IDGVTVARAISLKDKFENLGARLIQDVASKTNETAGDGTTTATV

LAKSIFSETVKNVAAGCNPMDLRRGTQAAVEAVVEFLQKNKRDITTSEEIAQVATISA

NGDTHIGKLIANAMEKVGKEGVITVKEGKTMEDELDITEGMRFDRGYVSPYFITDTKS

QKVEFEKPLILLSEKKISNVQDIIPALEASTQLRRPLVIIAEDIDGEALAVCILNKLR

GQLQVAAVKAPGFGDNR"

BASE COUNT 201 a 170 c 170 g 164 t

ORIGIN

1 atagatggtg taaccgttgc cagagctatt tccctcaagg acaaattcga gaatctcggt

61 gctagactta tccaagatgt tgcctcgaaa accaacgaga ccgctggtga tggaaccaca

121 accgctactg tccttgctaa atctattttc tccgagaccg taaagaacgt cgccgcagga

181 tgcaacccaa tggacttgcg cagaggtacc caagccgccg tggaggccgt tgttgagttt

241 ttgcaaaaga acaagcgtga tatcacaacc agcgaggaaa tcgcacaagt tgcgactatc

301 agtgcaaacg gtgataccca cattggaaaa ttgattgcca acgctatgga gaaggttgga

361 aaggaaggtg tcatcacagt caaggaagga aagaccatgg aggatgaact cgatattacc

421 gagggaatga gatttgaccg cggttatgtt tccccatact tcatcaccga taccaagtcg

481 caaaaggtgg aattcgagaa gccattgatt ctcctttctg agaagaagat ttcaaacgtc

541 caagatatta tcccagcact tgaggcgtct actcaacttc gccgtccttt ggtcatcatt

601 gctgaagata tcgatggaga agctctcgct gtatgcatcc ttaacaagct ccgtggtcaa

661 ctccaagttg ccgctgtcaa ggcccccggt ttcggtgata accga

//

LOCUS Seq2 705 bp DNA linear PLN 18-NOV-2022

DEFINITION Botrytis cinerea Bc.po03 HSP60 DNA, parcial CDS.

ACCESSION Seq2

VERSION

KEYWORDS .

SOURCE Botrytis cinerea (Botryotinia fuckeliana)

ORGANISM Botrytis cinerea

Eukaryota; Fungi; Dikarya; Ascomycota; Pezizomycotina;

Leotiomycetes; Helotiales; Sclerotiniaceae; Botrytis.

REFERENCE 1 (bases 1 to 705)

AUTHORS Castillo,L., Aguilera,L., Plaza,V., Olivares,L. and Marambio,B.

TITLE Direct Submission

JOURNAL Submitted (18-NOV-2022) Departamento de Biologia, Universidad de La

Serena, Raul Bitran 1305, La Serena, 1720256, Chile

COMMENT Bankit Comment: TOTAL # OF SEQS:3

##Assembly-Data-START##

Sequencing Technology :: Sanger dideoxy sequencing

##Assembly-Data-END##

FEATURES Location/Qualifiers

source 1..705

/organism="Botrytis cinerea"

/mol_type="genomic DNA"

/db_xref="taxon:40559"

gene <1..>705

/gene="HSP60"

CDS <1..>705

/gene="HSP60"

/codon_start=1

/product="heat shock protein 60"

/translation="IDGVTVARAISLKDKFENLGARLIQDVASKTNETAGDGTTTATV

LAKSIFSETVKNVAAGCNPMDLRRGTQAAVEAVVEFLQKNKRDITTSEEIAQVATISA

NGDTHIGKLIANAMEKVGKEGVITVKEGKTMEDELDITEGMRFDRGYVSPYFITDTKS

QKVEFEKPLILLSEKKISNVQDIIPALEASTQLRRPLVIIAEDIDGEALAVCILNKLR

GQLQVAAVKAPGFGDNR"

BASE COUNT 201 a 170 c 170 g 164 t

ORIGIN

1 atagatggtg taaccgttgc cagagctatt tccctcaagg acaaattcga gaatctcggt

61 gctagactta tccaagatgt tgcctcgaaa accaacgaga ccgctggtga tggaaccaca

121 accgctactg tccttgctaa atctattttc tccgagaccg taaagaacgt cgccgcagga

181 tgcaacccaa tggacttgcg cagaggtacc caagccgccg tggaggccgt tgttgagttt

241 ttgcaaaaga acaagcgtga tatcacaacc agcgaggaaa tcgcacaagt tgcgactatc

301 agtgcaaacg gtgataccca cattggaaaa ttgattgcca acgctatgga gaaggttgga

361 aaggaaggtg tcatcacagt caaggaagga aagaccatgg aggatgaact cgatattacc

421 gagggaatga gatttgaccg cggttatgtt tccccatact tcatcaccga taccaagtcg

481 caaaaggtgg aattcgagaa gccattgatt ctcctttctg agaagaagat ttcaaacgtc

541 caagatatta tcccagcact tgaggcgtct actcaacttc gccgtccttt ggtcatcatt

601 gctgaagata tcgatggaga agctctcgct gtatgcatcc ttaacaagct ccgtggtcaa

661 ctccaagttg ccgctgtcaa ggcccccggt ttcggtgata accga

//

LOCUS Seq3 705 bp DNA linear PLN 18-NOV-2022

DEFINITION Botrytis cinerea Bc.vi09 HSP60 DNA, parcial CDS.

ACCESSION Seq3

VERSION

KEYWORDS .

SOURCE Botrytis cinerea (Botryotinia fuckeliana)

ORGANISM Botrytis cinerea

Eukaryota; Fungi; Dikarya; Ascomycota; Pezizomycotina;

Leotiomycetes; Helotiales; Sclerotiniaceae; Botrytis.

REFERENCE 1 (bases 1 to 705)

AUTHORS Castillo,L., Aguilera,L., Plaza,V., Olivares,L. and Marambio,B.

TITLE Direct Submission

JOURNAL Submitted (18-NOV-2022) Departamento de Biologia, Universidad de La

Serena, Raul Bitran 1305, La Serena, 1720256, Chile

COMMENT Bankit Comment: TOTAL # OF SEQS:3

##Assembly-Data-START##

Sequencing Technology :: Sanger dideoxy sequencing

##Assembly-Data-END##

FEATURES Location/Qualifiers

source 1..705

/organism="Botrytis cinerea"

/mol_type="genomic DNA"

/db_xref="taxon:40559"

gene <1..>705

/gene="HSP60"

CDS <1..>705

/gene="HSP60"

/codon_start=1

/product="heat shock protein 60"

/translation="IDGVTVARAISLKDKFENLGARLIQDVASKTNETAGDGTTTATV

LAKSIFSETVKNVAAGCNPMDLRRGTQAAVEAVVEFLQKNKRDITTSEEIAQVATISA

NGDTHIGKLIANAMEKVGKEGVITVKEGKTMEDELDITEGMRFDRGYVSPYFITDTKS

QKVEFEKPLILLSEKKISNVQDIIPALEASTQLRRPLVIIAEDIDGEALAVCILNKLR

GQLQVAAVKAPGFGDNR"

BASE COUNT 204 a 168 c 169 g 164 t

ORIGIN

1 atagatggtg taaccgttgc cagagctatc tccctcaagg acaaattcga gaacctcggt

61 gctagactta tccaagatgt tgcctcgaaa accaacgaga ccgctggtga tggaaccaca

121 accgctactg tccttgctaa atctattttc tccgagaccg taaagaacgt cgccgcagga

181 tgcaacccaa tggacttgcg cagaggtacc caagctgccg tggaggccgt tgttgagttt

241 ttgcaaaaga acaagcgtga tatcacaaca agcgaggaaa tcgcacaagt tgcgactatc

301 agtgcaaacg gtgataccca catcggaaaa ttgattgcca acgctatgga aaaggttgga

361 aaggaaggtg ttatcacagt taaggaagga aagaccatgg aggacgaact cgatattacc

421 gagggaatga gatttgaccg cggttatgtt tccccatact tcatcaccga taccaagtcg

481 caaaaggtgg aattcgagaa gccattgatt ctcctttctg agaagaagat ttcaaacgtc

541 caagatatta tcccagcact tgaggcgtct actcaacttc gtcgtccttt ggtcatcatt

601 gctgaagata tcgatggaga agctctcgca gtatgcattc ttaacaagct ccgtggtcaa

661 ctccaagttg ccgctgtcaa ggcccccggt ttcggtgata accga

//

LOCUS Seq1 690 bp DNA linear PLN 18-NOV-2022

DEFINITION Botrytis cinerea Bc.ad03 G3PDH DNA, parcial CDS.

ACCESSION Seq1

VERSION

KEYWORDS .

SOURCE Botrytis cinerea (Botryotinia fuckeliana)

ORGANISM Botrytis cinerea

Eukaryota; Fungi; Dikarya; Ascomycota; Pezizomycotina;

Leotiomycetes; Helotiales; Sclerotiniaceae; Botrytis.

REFERENCE 1 (bases 1 to 690)

AUTHORS Castillo,L., Aguilera,L., Plaza,V., Olivares,L. and Marambio,B.

TITLE Direct Submission

JOURNAL Submitted (18-NOV-2022) Departamento de Biologia, Universidad de La

Serena, Raul Bitran 1305, La Serena, 1720256, Chile

COMMENT Bankit Comment: TOTAL # OF SEQS:3

##Assembly-Data-START##

Sequencing Technology :: Sanger dideoxy sequencing

##Assembly-Data-END##

FEATURES Location/Qualifiers

source 1..690

/organism="Botrytis cinerea"

/mol_type="genomic DNA"

/db_xref="taxon:40559"

gene <1..>690

/gene="G3PDH"

CDS <1..>690

/gene="G3PDH"

/codon_start=1

/product="glyceraldehyde 3 phosphate dehydrogenase"

/translation="AYMLKYDSTHGQFKGDIKVLADGLEVNGKKVKFYTERDPANIPW

AESEAYYVVESTGVFTTTEKAKAHLKGGAKKVVISAPSADAPMYVMGVNNETYTGDVD

VISNASCTTNCLAPLAKVINDEFTIIEGLMTTIHSYTATQKTVDGPSAKDWRGGRTAA

QNIIPSSTGAAKAVGKVIPVLNGKLTGMSMRVPTANVSVVDLTVRIEKGASYDEIKAV

IKKAADGPLKGK"

BASE COUNT 171 a 189 c 170 g 160 t

ORIGIN

1 gcatacatgt tgaagtatga ttccacccac ggtcaattca agggtgacat caaggtcctt

61 gccgatggat tggaggtcaa tggcaagaag gtcaagttct acaccgagag agacccagcc

121 aacatcccat gggctgagtc tgaggcatac tacgtcgtcg agtccaccgg tgttttcacc

181 accaccgaga aggccaaggc acatttgaag ggtggtgcca agaaggttgt tatctctgct

241 ccttctgccg atgccccaat gtacgttatg ggtgtcaaca acgagaccta cactggtgat

301 gttgatgtta tctccaacgc ctcttgcaca accaactgct tggctcctct cgccaaggtc

361 atcaacgatg agttcaccat cattgaaggt ttgatgacca ccatccactc ctacaccgct

421 acccaaaaga ccgttgatgg tccatccgct aaggattggc gtggaggacg taccgctgct

481 caaaacatca tcccatcgag caccggtgct gccaaggctg tcggaaaggt catcccagtc

541 cttaacggca aactcaccgg aatgtccatg cgtgttccaa ctgccaacgt ctcagttgtt

601 gacttgactg tccgcattga gaagggtgct tcttacgatg agatcaaggc cgtcatcaag

661 aaggctgctg atggtcctct caagggtaag

//

LOCUS Seq2 690 bp DNA linear PLN 18-NOV-2022

DEFINITION Botrytis cinerea Bc.po03 G3PDH DNA, parcial CDS.

ACCESSION Seq2

VERSION

KEYWORDS .

SOURCE Botrytis cinerea (Botryotinia fuckeliana)

ORGANISM Botrytis cinerea

Eukaryota; Fungi; Dikarya; Ascomycota; Pezizomycotina;

Leotiomycetes; Helotiales; Sclerotiniaceae; Botrytis.

REFERENCE 1 (bases 1 to 690)

AUTHORS Castillo,L., Aguilera,L., Plaza,V., Olivares,L. and Marambio,B.

TITLE Direct Submission

JOURNAL Submitted (18-NOV-2022) Departamento de Biologia, Universidad de La

Serena, Raul Bitran 1305, La Serena, 1720256, Chile

COMMENT Bankit Comment: TOTAL # OF SEQS:3

##Assembly-Data-START##

Sequencing Technology :: Sanger dideoxy sequencing

##Assembly-Data-END##

FEATURES Location/Qualifiers

source 1..690

/organism="Botrytis cinerea"

/mol_type="genomic DNA"

/db_xref="taxon:40559"

gene <1..>690

/gene="G3PDH"

CDS <1..>690

/gene="G3PDH"

/codon_start=1

/product="glyceraldehyde 3 phosphate dehydrogenase"

/translation="AYMLKYDSTHGQFKGDIKVLADGLEVNGKKVKFYTERDPANIPW

AESEAYYVVESTGVFTTTEKAKAHLKGGAKKVVISAPSADAPMYVMGVNNETYTGDVD

VISNASCTTNCLAPLAKVINDEFTIIEGLMTTIHSYTATQKTVDGPSAKDWRGGRTAA

QNIIPSSTGAAKAVGKVIPVLNGKLTGMSMRVPTANVSVVDLTVRIEKGASYDEIKAV

IKKAADGPLKGK"

BASE COUNT 171 a 189 c 170 g 160 t

ORIGIN

1 gcatacatgt tgaagtatga ttccacccac ggtcaattca agggtgacat caaggtcctt

61 gccgatggat tggaggtcaa tggcaagaag gtcaagttct acaccgagag agacccagcc

121 aacatcccat gggctgagtc tgaggcatac tacgtcgtcg agtccaccgg tgttttcacc

181 accaccgaga aggccaaggc acatttgaag ggtggtgcca agaaggttgt tatctctgct

241 ccttctgccg atgccccaat gtacgttatg ggtgtcaaca acgagaccta cactggtgat

301 gttgatgtta tctccaacgc ctcttgcaca accaactgct tggctcctct cgccaaggtc

361 atcaacgatg agttcaccat cattgaaggt ttgatgacca ccatccactc ctacaccgct

421 acccaaaaga ccgttgatgg tccatccgct aaggattggc gtggaggacg taccgctgct

481 caaaacatca tcccatcgag caccggtgct gccaaggctg tcggaaaggt catcccagtc

541 cttaacggca aactcaccgg aatgtccatg cgtgttccaa ctgccaacgt ctcagttgtt

601 gacttgactg tccgcattga gaagggtgct tcttacgatg agatcaaggc cgtcatcaag

661 aaggctgctg atggtcctct caagggtaag

//

LOCUS Seq3 690 bp DNA linear PLN 18-NOV-2022

DEFINITION Botrytis cinerea Bc.vi09 G3PDH DNA, parcial CDS.

ACCESSION Seq3

VERSION

KEYWORDS .

SOURCE Botrytis cinerea (Botryotinia fuckeliana)

ORGANISM Botrytis cinerea

Eukaryota; Fungi; Dikarya; Ascomycota; Pezizomycotina;

Leotiomycetes; Helotiales; Sclerotiniaceae; Botrytis.

REFERENCE 1 (bases 1 to 690)

AUTHORS Castillo,L., Aguilera,L., Plaza,V., Olivares,L. and Marambio,B.

TITLE Direct Submission

JOURNAL Submitted (18-NOV-2022) Departamento de Biologia, Universidad de La

Serena, Raul Bitran 1305, La Serena, 1720256, Chile

COMMENT Bankit Comment: TOTAL # OF SEQS:3

##Assembly-Data-START##

Sequencing Technology :: Sanger dideoxy sequencing

##Assembly-Data-END##

FEATURES Location/Qualifiers

source 1..690

/organism="Botrytis cinerea"

/mol_type="genomic DNA"

/db_xref="taxon:40559"

gene <1..>690

/gene="G3PDH"

CDS <1..>690

/gene="G3PDH"

/codon_start=1

/product="glyceraldehyde 3 phosphate dehydrogenase"

/translation="AYMLKYDSTHGQFKGDIKVLADGLEVNGKKVKFYTERDPANIPW

AESEAYYVVESTGVFTTTEKAKAHLKGGAKKVVISAPSADAPMYVMGVNNETYTGDVD

VISNASCTTNCLAPLAKVINDEFTIIEGLMTTIHSYTATQKTVDGPSAKDWRGGRTAA

QNIIPSSTGAAKAVGKVIPVLNGKLTGMSMRVPTANVSVVDLTVRIEKGASYDEIKAV

IKKAADGPLKGK"

BASE COUNT 171 a 189 c 170 g 160 t

ORIGIN

1 gcatacatgt tgaagtatga ttccacccac ggtcaattca agggtgacat caaggtcctt

61 gccgatggat tggaggtcaa tggcaagaag gtcaagttct acaccgagag agacccagcc

121 aacatcccat gggctgagtc tgaggcatac tacgtcgtcg agtccaccgg tgttttcacc

181 accaccgaga aggccaaggc acatttgaag ggtggtgcca agaaggttgt tatctctgct

241 ccttctgccg atgccccaat gtacgttatg ggtgtcaaca acgagaccta cactggtgat

301 gttgatgtta tctccaacgc ctcttgcaca accaactgct tggctcctct cgccaaggtc

361 atcaacgatg agttcaccat cattgaaggt ttgatgacca ccatccactc ctacaccgct

421 acccaaaaga ccgttgatgg tccatccgct aaggattggc gtggaggacg taccgctgct

481 caaaacatca tcccatcgag caccggtgct gccaaggctg tcggaaaggt catcccagtc

541 cttaacggca aactcaccgg aatgtccatg cgtgttccaa ctgccaacgt ctcagttgtt

601 gacttgactg tccgcattga gaagggtgct tcttacgatg agatcaaggc cgtcatcaag

661 aaggctgctg atggtcctct caagggtaag

//

LOCUS Seq1 336 bp DNA linear PLN 18-NOV-2022

DEFINITION Botrytis cinerea Bc.ad03 NET1 DNA, parcial CDS.

ACCESSION Seq1

VERSION

KEYWORDS .

SOURCE Botrytis cinerea (Botryotinia fuckeliana)

ORGANISM Botrytis cinerea

Eukaryota; Fungi; Dikarya; Ascomycota; Pezizomycotina;

Leotiomycetes; Helotiales; Sclerotiniaceae; Botrytis.

REFERENCE 1 (bases 1 to 336)

AUTHORS Castillo,L., Aguilera,L., Plaza,V., Olivares,L. and Marambio,B.

TITLE Direct Submission

JOURNAL Submitted (18-NOV-2022) Departamento de Biologia, Universidad de La

Serena, Raul Bitran 1305, La Serena, 1720256, Chile

COMMENT Bankit Comment: TOTAL # OF SEQS:3

##Assembly-Data-START##

Sequencing Technology :: Sanger dideoxy sequencing

##Assembly-Data-END##

FEATURES Location/Qualifiers

source 1..336

/organism="Botrytis cinerea"

/mol_type="genomic DNA"

/db_xref="taxon:40559"

gene <1..>336

/gene="NEP1"

CDS <1..>336

/gene="NEP1"

/codon_start=1

/product="necrosis and ethylene inducing protein 1"

/translation="DFPKDQPAAGNVVGGHRHDWEYVVAWVNNPEVANPTLIGAGASG

HGSIKKTTNPQRQGDRLKVEYYVSFPTNHELQFTNTLGRDLPMMWYDFLPAVSKTALQ

NTNFGKANCP"

BASE COUNT 92 a 103 c 79 g 62 t

ORIGIN

1 gacttcccca aggatcaacc agccgctgga aacgtcgtcg gaggtcaccg tcacgactgg

61 gagtatgtcg tcgcttgggt caacaacccc gaggttgcca accccacctt aatcggcgcc

121 ggcgcatccg gtcacggaag catcaagaag accacaaacc cccaacgcca gggcgacaga

181 ttgaaggtcg aatactatgt ctccttccca accaaccacg agttgcagtt caccaacacc

241 ttgggcagag atttgccaat gatgtggtat gacttcttgc cagctgttag caagacagct

301 ttacaaaaca ccaactttgg aaaggcaaac tgccca

//

LOCUS Seq2 336 bp DNA linear PLN 18-NOV-2022

DEFINITION Botrytis cinerea Bc.po03 NEP1 DNA, parcial CDS.

ACCESSION Seq2

VERSION

KEYWORDS .

SOURCE Botrytis cinerea (Botryotinia fuckeliana)

ORGANISM Botrytis cinerea

Eukaryota; Fungi; Dikarya; Ascomycota; Pezizomycotina;

Leotiomycetes; Helotiales; Sclerotiniaceae; Botrytis.

REFERENCE 1 (bases 1 to 336)

AUTHORS Castillo,L., Aguilera,L., Plaza,V., Olivares,L. and Marambio,B.

TITLE Direct Submission

JOURNAL Submitted (18-NOV-2022) Departamento de Biologia, Universidad de La

Serena, Raul Bitran 1305, La Serena, 1720256, Chile

COMMENT Bankit Comment: TOTAL # OF SEQS:3

##Assembly-Data-START##

Sequencing Technology :: Sanger dideoxy sequencing

##Assembly-Data-END##

FEATURES Location/Qualifiers

source 1..336

/organism="Botrytis cinerea"

/mol_type="genomic DNA"

/db_xref="taxon:40559"

gene <1..>336

/gene="NEP1"

CDS <1..>336

/gene="NEP1"

/codon_start=1

/product="necrosis and ethylene inducing protein 1"

/translation="DFPKDQPAAGNVVGGHRHDWEYVVAWVNNPEVANPTLIGAGASG

HGSIKKTTNPQRQGDRLKVEYYVSFPTNHELQFTNTLGRDLPMMWYDFLPAVSKTALQ

NTNFGKANCP"

BASE COUNT 92 a 103 c 79 g 62 t

ORIGIN

1 gacttcccca aggatcaacc agccgctgga aacgtcgtcg gaggtcaccg tcacgactgg

61 gagtatgtcg tcgcttgggt caacaacccc gaggttgcca accccacctt aatcggcgcc

121 ggcgcatccg gtcacggaag catcaagaag accacaaacc cccaacgcca gggcgacaga

181 ttgaaggtcg aatactatgt ctccttccca accaaccacg agttgcagtt caccaacacc

241 ttgggcagag atttgccaat gatgtggtat gacttcttgc cagctgttag caagacagct

301 ttacaaaaca ccaactttgg aaaggcaaac tgccca

//

LOCUS Seq3 336 bp DNA linear PLN 18-NOV-2022

DEFINITION Botrytis cinerea Bc.vi09 NEP1 DNA, parcial CDS.

ACCESSION Seq3

VERSION

KEYWORDS .

SOURCE Botrytis cinerea (Botryotinia fuckeliana)

ORGANISM Botrytis cinerea

Eukaryota; Fungi; Dikarya; Ascomycota; Pezizomycotina;

Leotiomycetes; Helotiales; Sclerotiniaceae; Botrytis.

REFERENCE 1 (bases 1 to 336)

AUTHORS Castillo,L., Aguilera,L., Plaza,V., Olivares,L. and Marambio,B.

TITLE Direct Submission

JOURNAL Submitted (18-NOV-2022) Departamento de Biologia, Universidad de La

Serena, Raul Bitran 1305, La Serena, 1720256, Chile

COMMENT Bankit Comment: TOTAL # OF SEQS:3

##Assembly-Data-START##

Sequencing Technology :: Sanger dideoxy sequencing

##Assembly-Data-END##

FEATURES Location/Qualifiers

source 1..336

/organism="Botrytis cinerea"

/mol_type="genomic DNA"

/db_xref="taxon:40559"

gene <1..>336

/gene="NEP1"

CDS <1..>336

/gene="NEP1"

/codon_start=1

/product="necrosis and ethylene inducing protein 1"

/translation="DFPKDQPAAGNVVGGHRHDWEYVVAWVNNPEVANPTLIGAGASG

HGSIKKTTNPQRQGDRLKVEYYVSFPTNHELQFTNTLGRDLPMMWYDFLPAVSKTALQ

NTNFGKANCP"

BASE COUNT 92 a 103 c 79 g 62 t

ORIGIN

1 gacttcccca aggatcaacc agccgctgga aacgtcgtcg gaggtcaccg tcacgactgg

61 gagtatgtcg tcgcttgggt caacaacccc gaggttgcca accccacctt aatcggcgcc

121 ggcgcatccg gtcacggaag catcaagaag accacaaacc cccaacgcca gggcgacaga

181 ttgaaggtcg aatactatgt ctccttccca accaaccacg agttgcagtt caccaacacc

241 ttgggcagag atttgccaat gatgtggtat gacttcttgc cagctgttag caagacagct

301 ttacaaaaca ccaactttgg aaaggcaaac tgccca

//
