# Supplementary figures and images for: Iron and copper on Botrytis cinerea: new inputs in the cellular characterization of their inhibitory effect
Source: PeerJ. 2023 Sep 20;11:e15994. doi: 10.7717/peerj.15994 (PMC10517660; doi:10.7717/peerj.15994)

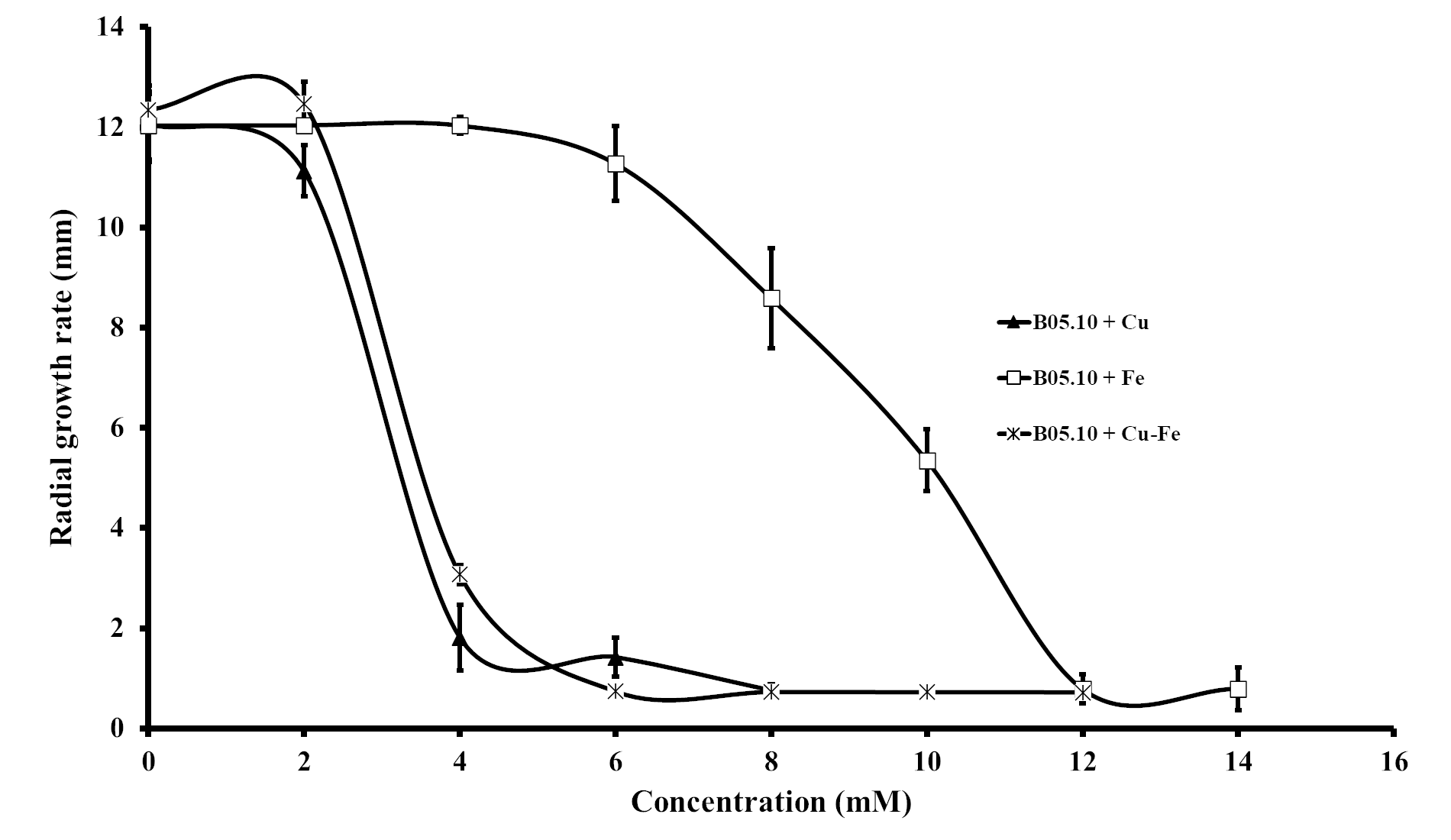

Supplement: Supplemental Information 3 [file peerj-11-15994-s003.png]

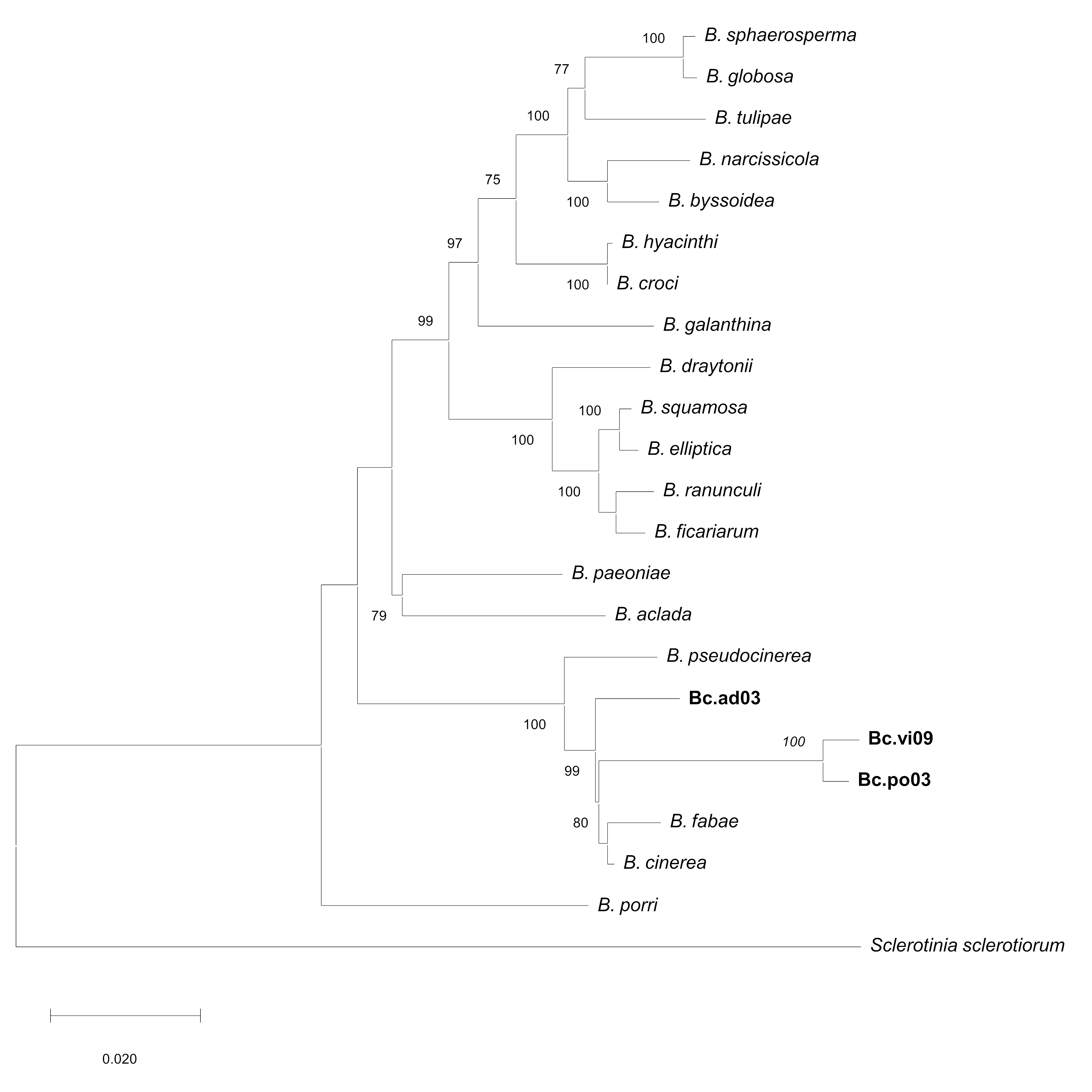

Supplement: Supplemental Information 4 — Sequences showing the phylogenetic position of native isolated of B. cinerea. Bootstrap values (>70%) based on 1,000 replications are shown at the nodes of the tree. Bar, 2 substitutions per 1,000 nt. Sclerotinia sclerotiorum was used as the outgroup. [file peerj-11-15994-s004.png]

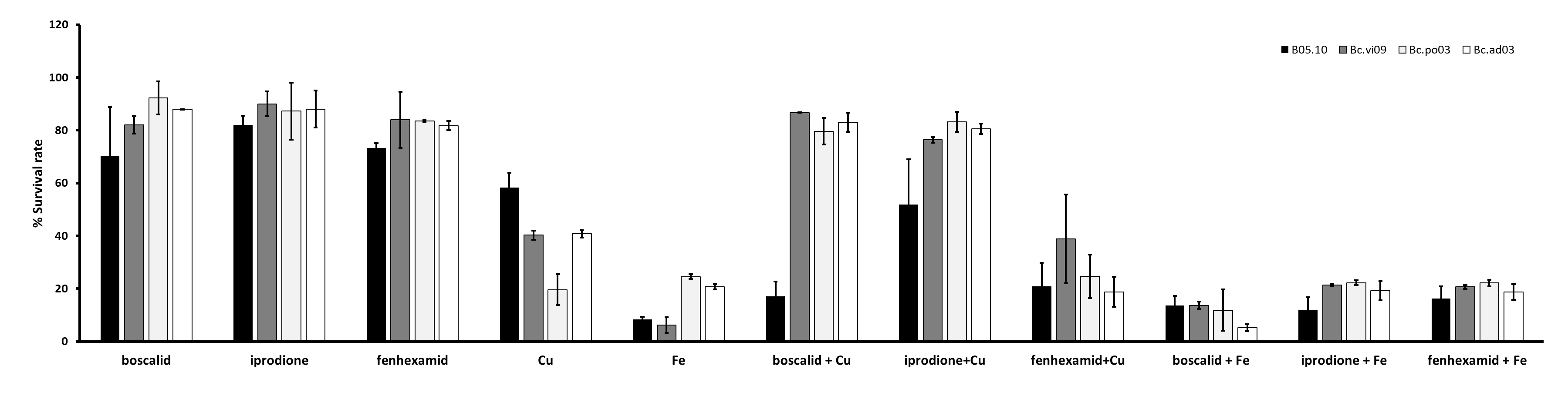

Supplement: Supplemental Information 5 [file peerj-11-15994-s005.png]
