# Supplementary material for: Iron and copper on Botrytis cinerea: new inputs in the cellular characterization of their inhibitory effect
Source: PeerJ. 2023 Sep 20;11:e15994. doi: 10.7717/peerj.15994 (PMC10517660; doi:10.7717/peerj.15994)
Supplement: Supplemental Information 6 [file peerj-11-15994-s006.docx]

**Table S1.** Characteristics of *B. cinerea* wild strains isolates from different plants and locations in Coquimbo Region, Chile

| Code | Plant name | Location  (In Coquimbo Region, Chile) | Genotype |
| --- | --- | --- | --- |
| Bc.po03 (19_12.2) | *Porlieria chilensis* | El Peñon | Boty |
| Bc.vi09 (20_25.6) | *Vitis vinifera* | Gualliguaica | Transposa |
| Bc.ad03 (22_11.1) | Unknown | Alcohuaz | Boty |
