# Supplementary material for: Iron and copper on Botrytis cinerea: new inputs in the cellular characterization of their inhibitory effect
Source: PeerJ. 2023 Sep 20;11:e15994. doi: 10.7717/peerj.15994 (PMC10517660; doi:10.7717/peerj.15994)
Supplement: Supplemental Information 7 [file peerj-11-15994-s007.docx]

**Table. S2**. Primers used in this study.

| **Name** | **Target region** | **Nucleotide sequence (5’ - 3’)** | **Reference** |
| --- | --- | --- | --- |
| ITS1 | 5.8SrDNA-ITS | TCCGTAGGTGAACCTGCGG | White *et al.* (1990) |
| ITS4 | 5.8SrDNA-ITS | TCCTCCGCTTATTGATATGC | White *et al.* (1990) |
| G3PDH-F | *G3PDH* | ATTGACATCGTCGCTGTCAACGA | Staats *et al.* (2005) |
| G3PDH-R | *G3PDH* | ACCCCACTCGTTGT CGTACCA | Staats *et al.* (2005) |
| HSP60-F | *HSP60* | CAACAATTGAGATTTGCCCACAAG | Staats *et al.* (2005) |
| HSP60-R | *HSP60* | GATGGATCCAGTGGTACCGAGCAT | Staats *et al.* (2005) |
| RPB2-F | *RPB2* | GATGATCGTGATCATTTCGG | Staats *et al.* (2005) |
| RPB2-R | *RPB2* | CCCATAGCTTGCTTACCCAT | Staats *et al.* (2005) |
| NEP1for | *NEP1* | CCAACGCAAAATTCCTTTCTATCC | Staats *et al.* (2005) |
| NEP1revB | *NEP1* | GTTGGCGAAGTTGTGGTCATTGAA | Staats *et al.* (2005) |
| Boty-F | Boty | TAACCTTGTCTTTGCTCATC | Ma and Michailides (2005) |
| Boty-R | Boty | CCCAATTTATTCAATGTCAG | Ma and Michailides (2005) |
| F300 | Flipper | GCACAAAACCTACAGAAGA | Ma and Michailides (2005) |
| F1500 | Flipper | ATTCGTTTCTTGGACTGTA | Ma and Michailides (2005) |
