# Supplementary material for: Iron and copper on Botrytis cinerea: new inputs in the cellular characterization of their inhibitory effect
Source: PeerJ. 2023 Sep 20;11:e15994. doi: 10.7717/peerj.15994 (PMC10517660; doi:10.7717/peerj.15994)
Supplement: Supplemental Information 8 [file peerj-11-15994-s008.docx]

**Table S3.** Patterns of sensitivity and resistance of three *B. cinerea* wild strains at certain concentrations to different commercial antifungals.

| Code | Tebuconazole (EC_50_ 1.28 µg/mL) | Timorex (EC_50_13.2 µg/mL) | Iprodione (EC_50_ 2 µg/mL) | Pyrimethanil (EC_50_ 12 µg/mL) | Boscalid (EC_50_ 6.4 µg/mL) | Fluodioxonil (EC_50_ 3.2 µg/mL) | Fenhexamid (EC_50_ 7.68 µg/mL) | Sanicitrex (EC_50_ 1400 µg/mL) | Bc-1000  (EC_50_ 35 µg/mL) |
| --- | --- | --- | --- | --- | --- | --- | --- | --- | --- |
| Bc.po03 | Sensitive | Resistant | Sensitive | Sensitive | Resistant | Sensitive | Sensitive | Sensitive | Resistant |
| Bc.vi09 | Sensitive | Sensitive | Sensitive | Sensitive | Sensitive | Sensitive | Sensitive | Sensitive | Resistant |
| Bc.ad03 | Sensitive | Sensitive | Sensitive | Sensitive | Sensitive | Sensitive | Resistant | Resistant | Resistant |

EC_50_: half maximal effective concentration (µg/mL)
