# Supplementary material for: Iron and copper on Botrytis cinerea: new inputs in the cellular characterization of their inhibitory effect
Source: PeerJ. 2023 Sep 20;11:e15994. doi: 10.7717/peerj.15994 (PMC10517660; doi:10.7717/peerj.15994)
Supplement: Supplemental Information 9 [file peerj-11-15994-s009.docx]

Table S4. GenBank accession numbers of native *B. cinerea* sequences, determined in this study.

|  | ***HSP60*** | ***G3PDH*** | ***RPB2*** | ***NEP1*** | ***ITS*** |
| --- | --- | --- | --- | --- | --- |
| Bc.ad03 | OP859134 | OP859137 | OP859131 | OP859140 | OP852422 |
| Bc.po03 | OP859135 | OP859138 | OP859132 | OP859141 | OP852423 |
| Bc.vi09 | OP859136 | OP859139 | OP859133 | OP859142 | OP852424 |
