# Supplementary material for: Iron and copper on Botrytis cinerea: new inputs in the cellular characterization of their inhibitory effect
Source: PeerJ. 2023 Sep 20;11:e15994. doi: 10.7717/peerj.15994 (PMC10517660; doi:10.7717/peerj.15994)
Supplement: Supplemental Information 10 — Percentage nucleotide identity of Bc.ad03, Bcpo03 and Bc.vi09 to B. cinerea B05.10. [file peerj-11-15994-s010.docx]

Table S5. Percentage nucleotide identity to *B. cinerea* B05.10.

|  | ***HSP60*** | ***G3PDH*** | ***RPB2*** | ***NEP1*** | ***ITS*** |
| --- | --- | --- | --- | --- | --- |
| Bc.ad03 | 100 | 100 | 100 | 100 | 98.28 |
| Bc.po03 | 100 | 100 | 100 | 100 | 99.60 |
| Bc.vi09 | 100 | 100 | 100 | 100 | 99.81 |
