# Supplementary material for: Iron and copper on Botrytis cinerea: new inputs in the cellular characterization of their inhibitory effect
Source: PeerJ. 2023 Sep 20;11:e15994. doi: 10.7717/peerj.15994 (PMC10517660; doi:10.7717/peerj.15994)
Supplement: Supplemental Information 11 [file peerj-11-15994-s011.docx]

**Table S6.** MI values for mixtures fungicides-metals on B05.10 and wild strains.

| Mixture index (MI) | B05.10 | Bc.vi09 | Bc.po03 | Bc.ad03 |
| --- | --- | --- | --- | --- |
| boscalid + Cu | 1.2 | 0.2 | 0.2 | 0.2 |
| iprodione + Cu | 0.8 | 0.3 | 0.2 | 0.3 |
| fenhexamid + Cu | 1.2 | 0.8 | 0.8 | 1.0 |
| boscalid + Fe | 0.7 | 0.8 | 1.1 | 1.0 |
| iprodione + Fe | 0.8 | 0.8 | 0.9 | 0.9 |
| fenhexamid + Fe | 0.7 | 0.7 | 0.8 | 0.8 |

synergism MI is >1, MI= 1 addition, and MI< 1 would be antagonism
